# Antiproliferative Activities and SwissADME Predictions of Physicochemical Properties of Carbonyl Group‐Modified Rotenone Analogues

**DOI:** 10.1002/open.202300087

**Published:** 2023-08-17

**Authors:** Rajelle D. Hernandez, Frances Abygail F. Genio, Jannelle R. Casanova, Marlon T. Conato, Monissa C. Paderes

**Affiliations:** ^1^ Institute of Chemistry University of the Philippines Diliman Quezon City Philippines 1101

**Keywords:** rotenone derivatives, anticancer, structure modification, natural products, antiproliferative

## Abstract

Rotenone is a naturally occurring compound shown to exhibit antiproliferative activity against various cancer cell lines, indicating its potential as a lead anticancer agent. However, its toxicity against normal cells has prompted further investigation and chemical modifications. In this study, a library of carbonyl group‐modified rotenone derivatives was synthesized and evaluated for their antiproliferative activities against MCF‐7 breast cancer cells, A549 human lung carcinoma cells, and HCT116 human colorectal cancer cells using 3‐(4, 5‐dimethylthiazolyl‐2)‐2, 5‐diphenyltetrazolium bromide (MTT) assay. The results showed several promising compounds that inhibited cell proliferation. Specifically, the oxime and alcohol rotenone derivatives exhibited antiproliferative activities against all 3 cancer cell lines, while the ethoxy, carbamate, and alkene derivatives are selective against MCF‐7 (IC_50_=5.72 μM), HCT116 (IC_50_=8.86 μM), and A549 (IC_50_=0.11 μM), respectively. SwissADME analysis showed that the physicochemical properties and drug‐likeness of the synthesized rotenone derivatives were within the set limits, suggesting the favorable characteristics of these compounds for drug development. The findings obtained in this work highlight the potential of rotenone derivatives as promising chemotherapeutic candidates.

## Graphical abstract

**Rotenone derivatives** were synthesized through modifications of the carbonyl group and their antiproliferative activities were evaluated against various cancer cell lines (MCF‐7, A549, and HCT116). The physicochemical properties and drug‐likeness of the derivatives were evaluated using the SwissADME webtool.

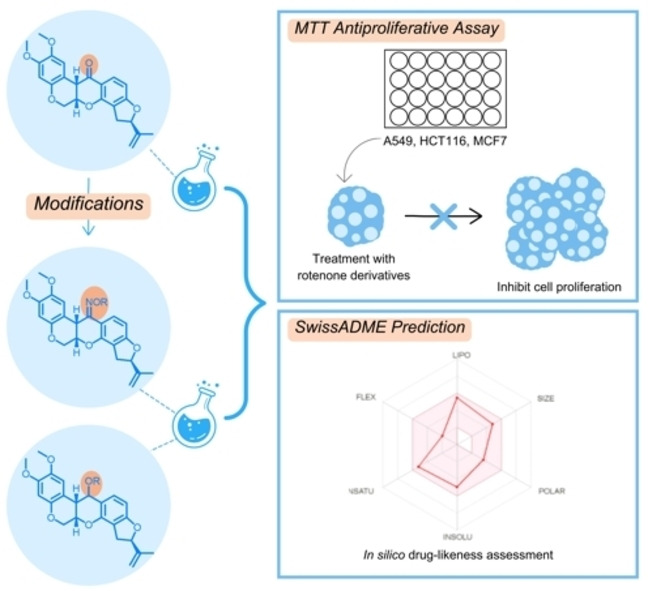

## Introduction

Cancer research has primarily focused on drug discovery and treatment development over the past few decades to combat the alarming rise in cancer‐related mortality. However, despite these efforts, cancer remains the second leading cause of death globally, with breast, lung, and colorectal cancer being the most prevalent types.^[1]^ Although chemotherapy is currently one of the most effective cancer treatments available, it often lacks specificity, leading to adverse and uncontrollable side effects. Additionally, cancer cells can develop resistance to these drugs, diminishing their effectiveness over time.^[2]^ The high cost of cancer drugs also presents a significant challenge for developing countries like the Philippines. Thus, it is crucial to continually search for and develop novel chemopreventive agents that exhibit high bioactivity, low toxicity, good pharmacokinetics, and are cost‐effective.

Natural products have proven to be a valuable source of biologically active compounds, particularly in the development of new anticancer drugs.^[3]^ However, their clinical applications are often hindered by limitations such as structural complexity, poor solubility, and metabolic instability. As a result, researchers have focused on structural modifications of natural products to enhance their efficacy, physicochemical properties, and pharmacokinetics, and expand their bioactivity spectrum.^[4]^

**Figure 1:**
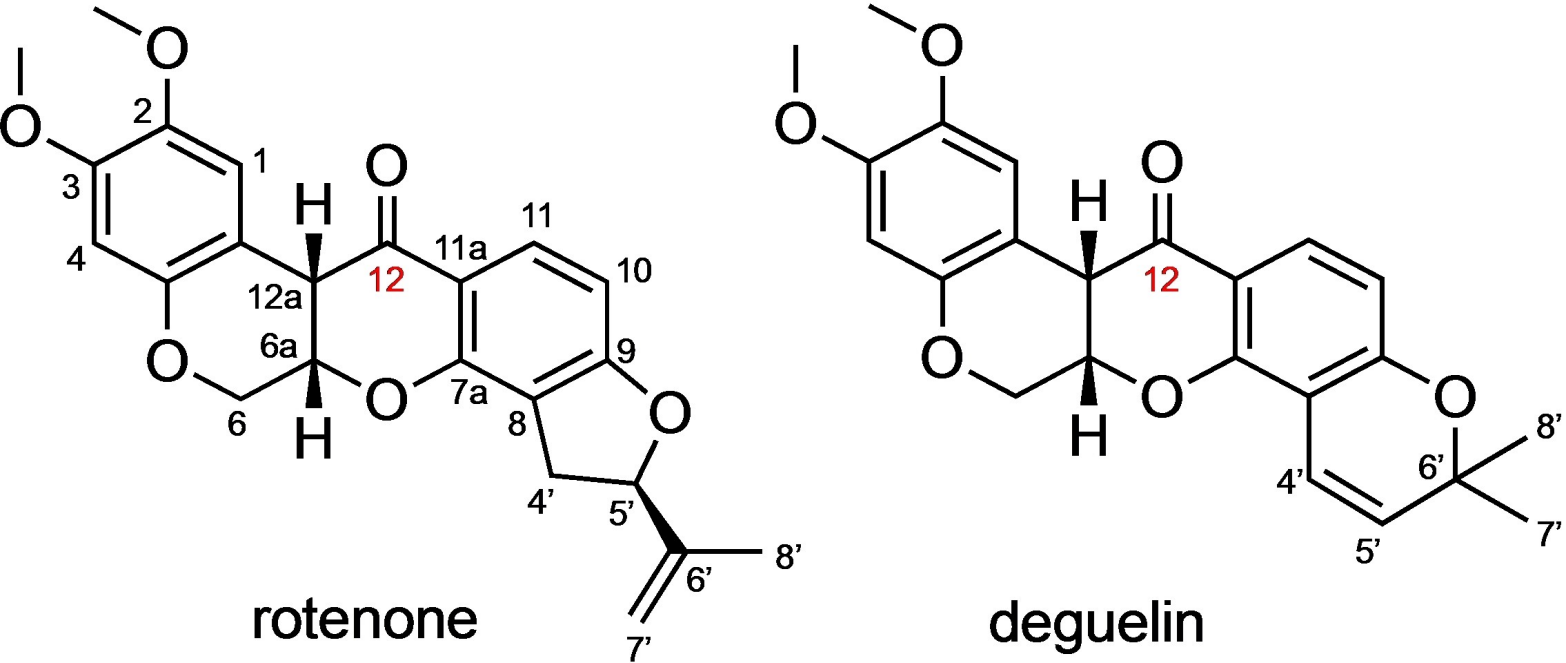
Structures of rotenone and deguelin.

Rotenoids are naturally occurring compounds^[5]^ known to possess cytotoxic properties against several cancer cell lines by targeting various cellular pathways.^[6]^ Among the rotenoids, the structural analogues, rotenone and deguelin (Figure 1) were the most extensively studied due to their potent anticancer properties.^[7]^ Studies indicate that rotenone impedes colon carcinoma cell proliferation by inhibiting PI3 K/AKT/mTOR signaling pathway.^[8]^ Activation of c‐Jun N‐terminal kinase (JNK) and p38 mitogen‐activated protein kinases (MAPKs), deactivation of extracellular signal‐regulated protein kinase 1/2 (ERK1/2), and increase in the reactive oxygen species production, leading to the accumulation of 4‐hydroxynonenal aggresomes and increase in autophagosomes, were some of the reported mechanisms by which rotenone induces apoptosis in cancer cells such as MCF‐7 breast cancer cells, and A549 human lung carcinoma cells.^[9]^ Despite their immense potential as anticancer drug candidates, they have been reported to be toxic to normal cells, which warrants chemical modifications to minimize toxicity and enhance their bioactivity.^[10]^ However, limited studies have been conducted especially on the chemical transformations of rotenone. In the study of Liu and co‐workers, spin‐labeled rotenone derivatives showed improved cytotoxicity against several cancer cell lines compared to the parent rotenone.^[11]^ A more recent study by Russell and co‐workers reported the synthesis and selective inhibition of hydroxylated rotenone and deguelin derivatives against prostate cancer cells.^[12]^ Because of the higher hydrophilicity of these compounds, they have lower probability of crossing the blood‐brain barrier, which reduces the risk of neurotoxicity. Furthermore, a comprehensive study on the structural modifications of deguelin was reported by Chang and co‐workers.^[13]^ Significant improvements in the antiproliferative activity against the H1299 non‐small‐cell lung cancer were observed with the carbonyl‐modified deguelin derivatives.

Inspired by the findings of Chang and colleagues,^[13]^ this study focuses on the C12 modifications of rotenone and evaluates their anticancer properties. Specifically, the preparation of carbonyl‐modified rotenone derivatives via incorporation of a range of functional groups was described. Their efficacy as antiproliferative agents against MCF‐7 breast cancer cells, A549 human lung carcinoma cells, and HCT116 human colorectal cancer cells were evaluated. Their physicochemical properties and drug‐likeness were predicted using the SwissADME tool, providing further insights into their potential as anticancer drugs.

## Results and Discussion

### Carbonyl group (C12) modifications of rotenone

**Scheme 1:**
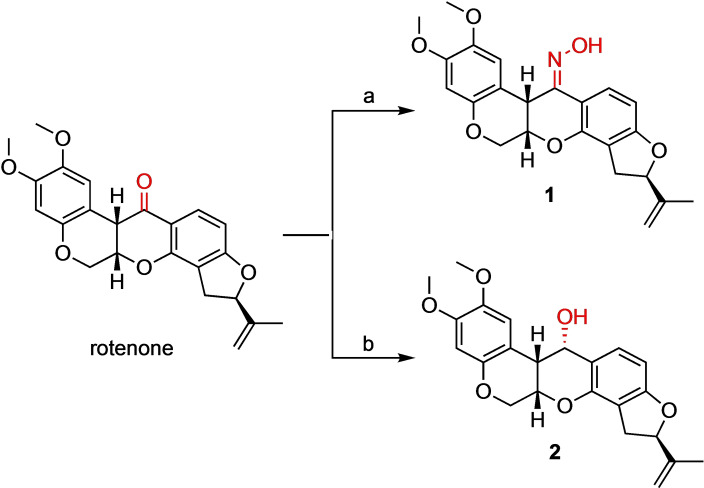
Synthesis of oxime and alcohol derivatives **1** and **2**. Reagents and conditions: (a) NH_2_OH⋅HCl, pyridine, 70 °C, 24 h, 96 %; (b) NaBH_4_, MeOH, 0 °C to rt, 3 h, 96 %.

**Scheme 2:**
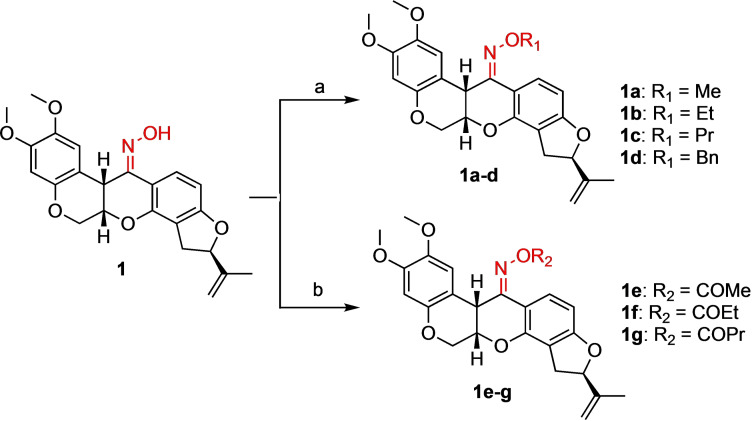
Synthesis of oxime ether and carbonyl oxime rotenone derivatives. Reagents and conditions: (a) *t*‐BuOK, THF, 0 °C, 2 h, **1 a**: CH_3_I, 74 %; **1 b**: CH_3_CH_2_I, 74 %; **1 c**: CH_3_CH_2_CH_2_I, 88 %; **1 d**: BnBr, 74 %, (b) Et_3_N, THF, 0 °C, 2 h, **1 e**: AcCl, 87 %; **1 f**: EtCOCl, 87 %; **1 g**: PrCOCl, 99 %.

**Scheme 3:**
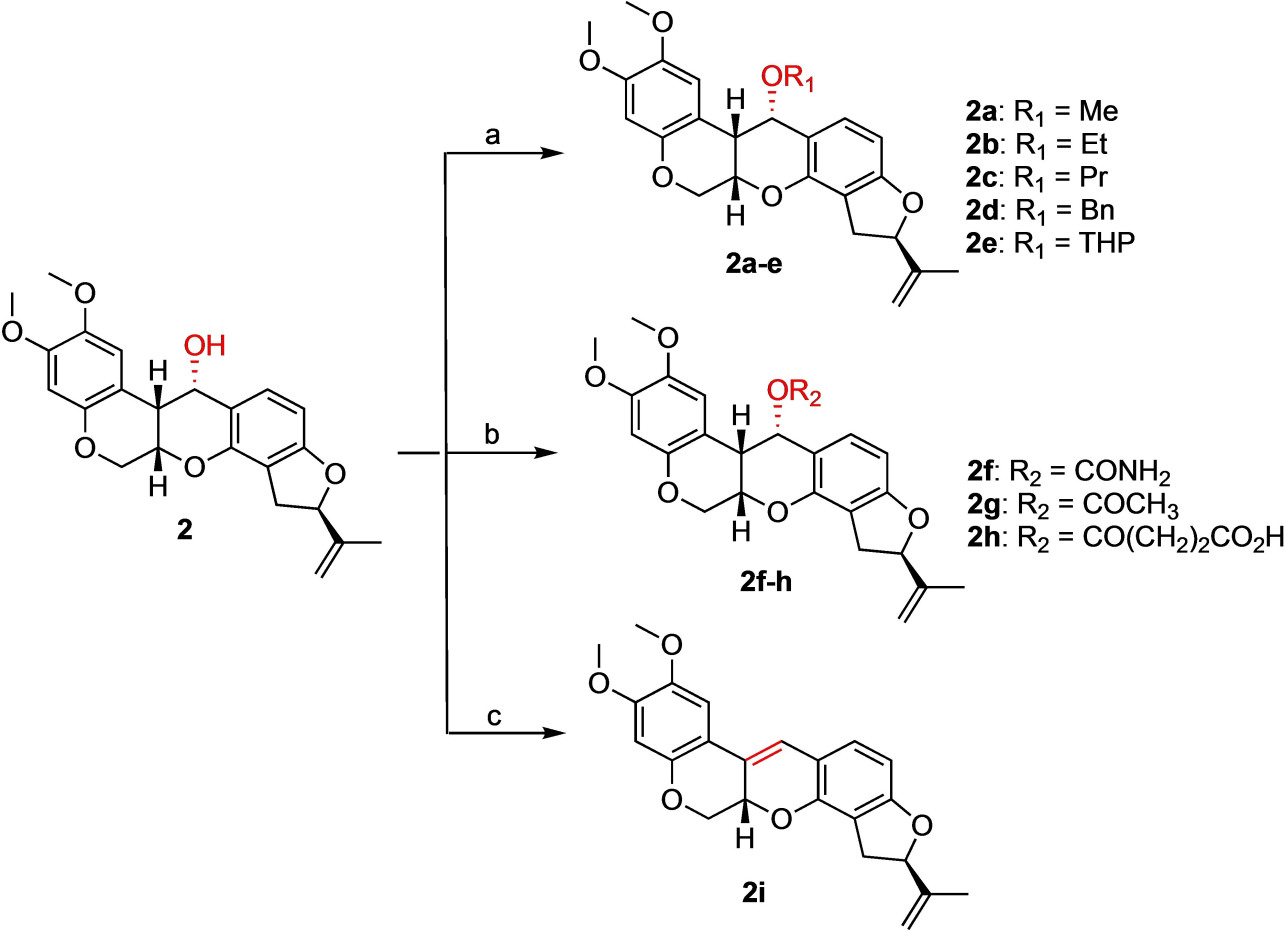
Derivatization of **2** by alkylation, acylation, and reduction to alkene. Reagents and conditions: (a) *t*‐BuOK, THF, 0 °C, **2 a**: CH_3_I, 2 h, 76 %; **2 b**: CH_3_CH_2_I, 24 h, 46 %; **2 c**: CH_3_CH_2_CH_2_I, 24 h, 87 %; **2 d**: BnBr, 24 h, 94 %; **2 e**: DHP, PPTS, CH_2_Cl_2_, rt, 1 h, 63 %, (b) **2 f**: CCl_3_CONCO, CH_2_Cl_2_, 0 °C, 30 mins, then MeOH/water, 2.5 h, 62 %; **2 g**: Ac_2_O, DMAP, Et_3_N, CH_2_Cl_2_, rt, 10 mins, 69 %; **2 h**: succinic anhydride, DMAP, Et_3_N, CH_2_Cl_2_, rt, 24 h, 79 %. (c) AcOH, 100 °C, 2 h, 100 %.

Carbonyl‐modified rotenone analogues were synthesized following the synthetic strategies outlined in Schemes 1, 2, 3. Oxime (**1**) and alcohol (**2**) derivatives were initially synthesized from commercially available rotenone (Scheme 1).[^13^, ^14^] Rotenone was quantitatively converted to compound **1** using hydroxylamine hydrochloride and pyridine. The stereospecific reduction of the carbonyl group of rotenone using sodium borohydride in methanol produced compound **2** in 96 % yield. The stereoselectivity of the reaction is attributed to the attack on the butterfly wing conformer of rotenone, in which the hydride delivery took place on the more accessible side of the molecule.^[15]^

Compound **1** was further derivatized either by alkylation or esterification (Scheme 2). The introduction of alkyl substituents to oxime derivative **1** was performed using alkyl halides and potassium *tert*‐butoxide in tetrahydrofuran (THF) to provide oxime ether rotenone derivatives **1 a**–**d**. Treatment with acyl chlorides and triethylamine (Et_3_N) in THF produced carbonyl oxime derivatives **1 e**–**g** with excellent yields.

Compounds **2 a**–**h** were synthesized from alcohol derivative **2** following the procedures shown in Scheme 3. Similar oxime alkylation conditions were used for the alcohol analogue **2**, where it was treated with the corresponding alkyl halides and potassium *tert*‐butoxide to provide the corresponding alkyl ether rotenone derivatives **2 a**–**d**. The alkyl ether **2 e** was produced via acid catalyzed THP protection of the C12 hydroxyl group of **2**. Carbamoylation of **2** with trichloroacetyl isocyanate followed by hydrolysis with methanol/water mixture afforded **2 f**. Treatment of **2** with acid anhydrides in the presence of DMAP and Et_3_N yielded **2 g**–**h**. Dehydration of **2** in glacial acetic acid at 100 °C yielded **2 i** with excellent yield.

### Inhibitory activities against MCF‐7, A549, and HCT116 cancer cells

The inhibitory activities of the rotenone derivatives against breast, lung, and colorectal cancer cells were evaluated using MTT reduction assay. MTT assay is the most widely used screening method to analyze cell proliferation. A range of concentrations (0.78 to 100 μg/mL) of the rotenone analogues was tested, and dose‐response curves were generated. The half‐maximal inhibitory concentrations (IC_50_) of the analogues were calculated for each cell line (Table 1). The IC_50_ values for the parent compound, rotenone, were also determined. Compounds with an IC_50_ value of less than 10 μM were deemed active.^[16]^

**Table 1:** IC_50_ values of rotenone derivatives against MCF‐7, A549, and HCT116 cancer cells.

Compound	IC_50_ (μM)^[a]^		
	MCF‐7	A549	HCT 116
Doxorubicin	5.89	7.36	0.21
Rotenone	1.98	>100	2.16
**1**	1.91	0.28	0.72
**1 a**	n.d. ^[b]^	>100	>100
**1 b**	n.d.	52.4	17.7
**1 c**	>100	>100	>100
**1 d**	16.2	36.1	n.d.
**1 e**	69.5	>100	48.3
**1 f**	46.4	38.9	25.7
**1 g**	66.9	81.0	n.d.
**2**	1.97	0.45	3.99
**2 a**	11.4	>100	>100
**2 b**	5.72	66.4	43.2
**2 c**	54.8	>100	>100
**2 d**	84.3	95.8	>100
**2 e**	27.8	30.9	>100
**2 f**	>100	>100	8.86
**2 g**	n.d.	>100	>100
**2 h**	n.d.	>100	>100
**2 i**	69.2	0.11	43.3

^[a]^IC_50_ values were determined as the mean of triplicate experiments. ^[b]^IC_50_ values were not determined (n.d.) due to low inhibition or compounds did not follow a dose‐response curve (i.e., the percent inhibition plateaued at around 40–60 % at all concentrations tested).

The MTT assay revealed that the rotenone derivatives exhibited different growth responses against MCF‐7, A549, and HCT116 cell lines (Table 1). When tested against MCF‐7 cells, compounds **1**, **2**, and **2 b** were found to exhibit cytotoxicity. Compounds **1** and **2** showed higher potency than doxorubicin (IC_50_=5.89 μM) and comparable activity with the parent compound rotenone (IC_50_=1.98 μM). It is noteworthy that the cytotoxic property of rotenone against MCF‐7 is consistent with those previously determined IC_50_ values.^[6b]^

In the antiproliferative assay against A549 cells (Table 1), three analogues (**1**, **2**, and **2 i**) were found to be more potent than rotenone. The introduction of oxime group (**1**), reduction of the carbonyl group to alcohol (**2**), and alkene (**2 i**) caused significant increase in activity. Their IC_50_ values are 0.28 μM, 0.45 μM, and 0.11 μM, respectively. Previous studies showed that rotenone extracted from *Neurautanenia mitis* exhibited cytotoxicity against A549 cells with an IC_50_ value of 25 nM.^[6b]^ However, in this study, rotenone did not exhibit cytotoxicity (IC_50_ >100 μM). The discrepancy between these findings may be due to the limitations of the MTT assay, which only assesses cell proliferation and viability. It is possible that rotenone‘s activity against A549 cells may be attributed to another cancer hallmark, or other biological pathways such as apoptosis, migration, or angiogenesis.

Both the oxime derivative **1** and the alcohol analogue **2** exhibit potency against HCT116 cells. Compound **1** showed a 3‐fold increase in activity when compared with rotenone, while the activity of compound **2** is lower by 2‐fold (Table 1). The carbamate derivative **2 f** also exhibited activity against HCT116 cells, with an IC_50_ value of 8.85 μM.

Generally, the oxime and alcohol derivatives **1** and **2** showed high inhibitory activities against the three cancer cell lines used. Introduction of bulky substituents to both **1** and **2** resulted in a decrease or complete loss of efficacy against the cell lines tested. This suggests that the bulky substituents may hinder the binding of rotenone to cellular receptors that are essential for inhibiting cell proliferation.

### Theoretical prediction of ADMET parameters using SwissADME webtool

Evaluation of pharmacokinetic profile of a molecule is a crucial aspect in drug discovery and development, which includes parameters related to its absorption, distribution, metabolism, excretion, and toxicity (ADMET).^[17]^ In this study, SwissADME, a free and readily accessible webtool was used to predict the physicochemical properties, pharmacokinetics, and drug‐likeness of the synthesized rotenone derivatives.^[18]^ 2D structures were imported into the website's interface in canonical simplified molecular‐input line‐entry system (SMILES) format and the ADMET properties were generated.

The ability of a drug to penetrate membranes for transport throughout the body is highly correlated with its physicochemical properties.^[19]^ Six (6) properties were considered which includes lipophilicity (XLOGP3 between −0.7 and+5.0), size (MW between 150 and 500 g/mol), polarity (topological polar surface area, TPSA between 20 and 130 Å^2^), solubility (log *S* ≤ 6), saturation (fraction of carbons in the sp^3^ hybridization ≥0.25), and flexibility (rotatable bonds ≤ 9).^[18]^ Table 2 presents the physicochemical properties of rotenone and its derivatives. Most compounds, including bioactive ones (**1**, **2**, **2 b**, **2 f**, and **2 i**) exhibited optimal values for all six properties, indicating good oral bioavailability.^[20]^ However, some derivatives have high lipophilicity (XLOGP3>5), and this is often associated with poor absorption,^[21]^ low solubility,^[22]^ in vivo toxicity,^[23]^ and reduced in vitro receptor selectivity.^[24]^ In most cases, high lipophilicity would lead to the exclusion of a molecule as a potential drug candidate.

**Table 2:** Calculated physicochemical parameters of rotenone and its derivatives^[a]^.

Molecule	XLOGP3	MW	TPSA	ESOL Log S	Fraction C−sp^3^	RB
Rotenone	4.1	394.42	63.22	−4.98	0.35	3
**1**	4.36	409.43	78.74	−5.22	0.35	3
**1 a**	4.68	423.46	67.74	−5.44	0.38	4
**1 b**	5.05	437.48	67.74	−5.68	0.4	5
**1 c**	5.58	451.51	67.74	−6.03	0.42	6
**1 d**	6.18	499.55	67.74	−6.79	0.3	6
**1 e**	4.46	451.47	84.81	−5.39	0.36	5
**1 f**	4.93	465.5	84.81	−5.7	0.38	6
**1 g**	5.29	479.52	84.81	−5.94	0.41	7
**2**	3.63	396.43	66.38	−4.69	0.39	3
**2 a**	4.16	410.46	55.38	−5.04	0.42	4
**2 b**	4.53	424.49	55.38	−5.28	0.44	5
**2 c**	5.06	438.51	55.38	−5.63	0.46	6
**2 d**	5.66	486.56	55.38	−6.4	0.33	6
**2 e**	5.02	480.55	64.61	−5.91	0.5	5
**2 f**	3.63	439.46	98.47	−4.8	0.38	5
**2 g**	4.20	438.47	72.45	−5.15	0.4	5
**2 h**	3.63	496.51	109.75	−4.92	0.41	8
**2 i**	4.46	378.42	46.15	−5.12	0.3	3

^[a]^XLOGP3 (between −0.7 and+5.0), MW (between 150 and 500 g/mol), TPSA (between 20 and 130 Å^2^), log *S* (≤6), fraction of carbons in the sp^3^ hybridization (≥0.25), rotatable bonds (RB≤9)

SwissADME can also predict the gastrointestinal (GI) absorption and brain penetration, two pharmacokinetic behaviors linked with lipophilicity and polarity of the molecules.^[25]^ As shown in Table S4 (Supporting Information), all of the synthesized rotenone derivatives were predicted to have high level of GI absorption. Moreover, the analysis provided insights into the probability of some compounds crossing the blood‐brain barrier (BBB), although experimental studies are necessary to validate these predictions. Interestingly, the results indicated that almost all derivatives are not P‐glycoprotein (P‐gp) inhibitors, implying that they are unlikely to be substrates for the efflux pump and may exhibit lower potential for drug‐drug interactions.^[26]^

Drug‐likeness is an essential aspect of drug development that evaluates the potential of a molecule to become an oral drug.^[27]^ To evaluate the drug‐likeness of the rotenone derivatives, five methods were used, which include the Lipinski (Pfizer), Ghose (Amgen), Veber (GSK), Egan (Pharmacia) and Muegge (Bayer) (Table S5, Supporting Information). Each method considers a range of physicochemical properties that define a molecule as drug‐like. Lipinski's rule of five is the pioneer filter implemented and is widely used,^[22]^ but the best method is dependent on the specific needs. The compounds analyzed in this study showed no violations in Lipinski's filter, and only minimal violations were observed in the other filters (Table S5 Supporting Information). To assess the likelihood of false‐positive results in assays, PAINS (Pan Assay Interference Compounds) score was used to evaluate common substructural motifs.^[28]^ Although no alerts were observed in the PAINS scores for all the derivatives, orthogonal experiments are recommended to validate the results.

## Conclusions

In this work, carbonyl group‐modified rotenone derivatives were synthesized and evaluated for their antiproliferative activities against MCF‐7 breast cancer cells, A549 human lung carcinoma cells, and HCT116 human colorectal cancer cells using MTT assay. Among the derivatives, five (5) promising compounds (**1**, **2**, **2 b**, **2 f**, and **2 i**) were identified with significant inhibitory effects on cell growth. The oxime and alcohol derivatives **1** and **2** exhibited potent activity against all the tested cancer cell lines, while compounds **2 b**, **2 f**, and **2 i** showed selective inhibitory activity against MCF‐7, HCT116 and A549 cancer cells, respectively. The addition of bulky substituents to compounds **1** and **2** led to a reduction or complete loss of effectiveness against the tested cell lines. SwissADME analysis predicted acceptable physicochemical and drug‐like characteristics for all the bioactive compounds, but further studies are necessary to validate these predictions. The identified derivatives hold great promise as potential therapeutic agents for cancer treatment and may be subjected to further biological evaluations using advanced cell‐based assays, metabolism‐based research, and in vivo experiments in various animal models. Further optimization of the chemical structures of the rotenone derivatives is also currently underway to enhance their inhibitory activity against cancer cells.

## Experimental Section

**General**: All reagents were purchased from Sigma‐Aldrich and TCI Chemicals used as received without further purification unless otherwise specified. ^1^H and ^13^C NMR spectra were recorded using Varian 500 MHz spectrometer with either CDCl_3_ or DMSO‐*d_6_* as solvent. NMR spectra were reported in ppm using residual chloroform (7.26 ppm for ^1^H NMR and 77.16 ppm for ^13^C NMR) or DMSO (2.50 ppm for ^1^H NMR and 39.52 ppm for ^13^C NMR) as reference peak. FTIR spectra were measured using Shimadzu IR Prestige 21 Fourier Transform Infrared spectrophotometer. High resolution mass spectra were obtained using Waters Acquity UPLC H‐Class‐Xevo G2XS Quadrupole Time‐of‐Flight High Resolution Mass Spectrometer in positive ion mode. Melting points of the solid compounds were measured using Cole Parmer Electrochemical IA9200. The purity of all synthesized derivatives was evaluated by HPLC analysis (≥95 %) using Shimadzu LC‐20 AD Prominence Liquid Chromatograph equipped with Shimadzu SPD−M20A prominence photodiode array detector.

### Experimental Procedures

**(6a*R*,12a*S*,E)‐8,9‐dimethoxy‐2‐(prop‐1‐en‐2‐yl)‐1,2,6 a,12,12 a,13 a‐hexahydrochromeno[3,4‐b]furo[2,3‐h]chromen‐6(5aH)‐one oxime (1)**. To a solution of rotenone (1.0 g, 0.06 mmol, 1 equiv) in pyridine (40 mL) was added hydroxylamine hydrochloride (0.88 g, 0.30 mmol, 5 equiv). The reaction mixture was heated to 70 °C and stirred overnight. The resulting mixture was quenched with cold water (20 mL) and filtered with nylon filter (0.2 μm). The collected precipitate was washed with cold ethanol and cold water alternately (3x). The rotenone oxime **1** was dried *in vacuo* and obtained as white powder (1.0 g, 96 % yield). The following data matched the reported characterization data:^[14a]^ ^1^H NMR (500 MHz, CDCl_3_). δ 7.69 (d, J=8.5 Hz, 1H), 6.62 (s, 1H), 6.45 (s, 1H), 6.43 (s, 1H), 5.15 (t, J=8.9 Hz, 1H), 5.06 (s, 1H), 4.90 (s, 1H), 4.87 (d, J=3.1 Hz, 1H), 4.61 (dd, J=12.0, 1.9 Hz, 1H), 4.53 (s, 1H), 4.26 (d, J=12.0 Hz, 1H), 3.80 (s, 3H), 3.74 (s, 3H), 3.29 (dd, J=15.7, 9.8 Hz, 1H), 2.93 (dd, J=15.7, 8.2 Hz, 1H), 1.76 (s, 3H). ^13^C NMR (126 MHz, CDCl_3_) δ 163.3, 152.7, 151.8, 149.4, 147.8, 143.8, 143.7, 125.4, 113.4, 112.3, 112, 108.6, 106.4, 104.2, 100.7, 87.1, 69.6, 67.1, 56.6, 56.0, 31.9, 31.8, 17.3. FTIR (ATR‐FTIR) 3465, 3083, 2966, 2926, 2852, 2827, 1686, 1609, 1505, 1456, 1407. HRMS (ESI) calcd for [M+H]^+^ C_23_H_24_NO_6:_ 410.1604, found: 410.1617.

**General procedure for the preparation of compounds 1 a–1 d**. To a mixture of alkyl halide (0.7 mmol, 1 equiv) and potassium *tert*‐butoxide (0.15 mmol, 2 equiv) in THF (1.0 mL), was added dropwise a solution of 100 mg of **1** (0.07 mmol) in THF. The reaction mixture was stirred for 2 h in an ice bath. The reaction was quenched with ammonium chloride (0.5 mL) and the resulting mixture extracted with DCM. The organic layer was dried over MgSO_4_ and concentrated under reduced pressure.

**(6a*R*,12a*S*)‐8,9‐dimethoxy‐2‐(prop‐1‐en‐2‐yl)‐1,2,12,12 a‐tetrahydrochromeno[3,4‐b]furo[2,3‐h]chromen‐6(6aH)‐one O‐methyl oxime (1 a)**. The residue was purified by flash column chromatography on silica gel (EtOAc/*n*‐hexane=1 : 3) to afford methyl oxime **1 a** as white solid powder (23 mg, 74 % yield). The obtained methyl oxime derivative matched the reported ^1^H NMR data.^[14a]^ ^1^H NMR (500 MHz, CDCl_3_) δ 7.77 (d, *J*=8.5 Hz, 1H), 6.52 (s, 1H), 6.43 (d, *J*=8.7 Hz, 1H), 6.41 (s, 1H), 5.13 (t, *J*=8.9 Hz, 1H), 5.05 (s, 1H), 4.89 (s, 1H), 4.75 (s, 1H), 4.58 (d, *J*=12.1 Hz, 1H), 4.49 (s, 1H), 4.21 (d, *J*=12.1 Hz, 1H), 4.05 (s, 3H), 3.79 (s, 3H), 3.75 (s, 3H), 3.27 (dd, *J*=15.6, 9.8 Hz, 1H), 2.91 (dd, *J*=15.7, 8.1 Hz, 1H), 1.75 (s, 3H). HRMS (ESI) calcd for [M]^+^ C_24_H_25_NO_6_: 423.1682, found: 423.2752.

**(6a*R*,12a*S*)‐8,9‐dimethoxy‐2‐(prop‐1‐en‐2‐yl)‐1,2,12,12 a‐tetrahydrochromeno[3,4‐b]furo[2,3‐h]chromen‐6(6aH)‐one O‐ethyl oxime (1 b** *)*. The residue was purified by flash column chromatography on silica gel (EtOAc/*n*‐hexane=1 : 3) to afford ethyl oxime **1 b** as a white solid powder (79 mg, 74 % yield). The obtained ethyl oxime derivative matched the reported characterization data.^[14a]^ ^1^H NMR (500 MHz, CDCl_3_) δ 7.78 (d, *J*=8.5 Hz, 1H), 6.56 (s, 1H), 6.44 (s, 1H), 6.42 (s, 1H), 5.14 (t, *J*=8.9 Hz, 1H), 5.05 (s, 1H), 4.89 (s, 1H), 4.78 (s, 1H), 4.59 (d, *J*=12.0 Hz, 1H), 4.50 (s, 1H), 4.31 (q, 2H), 4.23 (d, *J*=12.0 Hz, 1H), 3.79 (s, 3H), 3.74 (s, 3H), 3.28 (dd, *J*=15.6, 9.8 Hz 1H), 2.90 (dd, *J*=15.6, 9.8 Hz, 1H), 1.75 (s, 3H), 1.39 (t, *J*=6.9 Hz, 3H). ^13^C NMR (126 MHz, CDCl_3_) δ=163.0, 152.4, 149.6, 149.2, 147.7, 143.7, 143.6, 125.4, 113.1, 112.1, 111.7, 108.9, 106.6, 103.9, 100.6, 86.9, 69.9, 69.5, 67.0, 56.3, 55.9, 32.4, 31.9, 17.2, 15.0. HRMS (ESI) calcd for [M]^+^ C_25_H_27_NO_6_: 437.1838, found: 437.2001.

**(6a*R*,12a*S*)‐8,9‐dimethoxy‐2‐(prop‐1‐en‐2‐yl)‐1,2,12,12 a‐tetrahydrochromeno[3,4‐b]furo[2,3‐h]chromen‐6(6aH)‐one O‐propyl oxime (1 c)**. The residue was purified by flash column chromatography on silica gel (EtOAc/*n*‐hexane=1 : 3) to afford a white solid powder of propyl oxime **1 c** (97 mg, 88 % yield). The propyl oxime derivative matched the reported ^1^H NMR data.^[14a]^ ^1^H NMR (500 MHz, CDCl_3_) δ 7.76 (d, *J*=8.6 Hz, 1H), 6.55 (s, 1H), 6.44 (s, 1H), 6.42 (s, 1H), 5.14 (s, 1H), 5.05 (s, 1H), 4.89 (s, 1H), 4.78 (s, 1H), 4.59 (d, *J*=12.0 Hz, 1H), 4.50 (s, 1H), 4.24 (d, *J*=12.4 Hz, 1H), 4.24–4.19 (m, 2H), 3.79 (s, 3H), 3.74 (s, 3H), 3.26 (d, *J*=9.8 Hz, 1H), 2.93 (d, *J*=8.1 Hz, 1H), 1.85–1.76 (m, 2H), 1.75 (s, 3H), 1.01 (t, *J*=7.4 Hz, 3H). HRMS (ESI) calcd for [M]^+^ C_26_H_29_NO_6_: 451.1995, found: 451.2056.

**(6a*R*,12a*S*)‐8,9‐dimethoxy‐2‐(prop‐1‐en‐2‐yl)‐1,2,12,12 a‐tetrahydrochromeno[3,4‐b]furo[2,3‐h]chromen‐6(6aH)‐one O‐benzyl oxime (1 d)**. The residue was purified by column chromatography on silica gel (EtOAc/*n*‐hexane=1 : 3) to afford a white solid powder (90 mg, 74 % yield). The benzyl oxime derivative **1 d** matched the reported ^1^H NMR data.^[14b]^ ^1^H NMR (500 MHz, CDCl_3_) δ 7.80 (d, *J*=8.6 Hz, 1H), 7.54–7.45 (m, 2H), 7.48–7.35 (m, 3H), 6.47 (s, 1H), 6.45 (d, *J*=8.6 Hz, 1H), 6.40 (s, 1H), 5.29 (dd, *J*=50.3, 12.0 Hz, 2H), 5.15 (t, *J*=8.9 Hz, 1H), 5.06 (s, 1H), 4.90 (s, 1H), 4.80 (d, *J*=3.2 Hz, 1H), 4.57 (dd, *J*=12.0, 2.3 Hz, 1H), 4.49 (d, *J*=2.6 Hz, 1H), 4.21 (d, *J*=11.9 Hz, 1H), 3.78 (s, 3H), 3.51 (s, 3H), 3.28 (dd, *J*=15.7, 9.8 Hz, 1H), 2.92 (dd, *J*=15.7, 8.2 Hz, 1H), 1.76 (s, 3H). ^13^C NMR (126 MHz, CDCl_3_) δ=163.1, 152.5, 150.2, 149.1, 149.1, 147.5, 143.6, 137.5, 128.7, 128.5, 128.1, 125.4, 113.1, 112.1, 111.5, 108.7, 106.4, 103.9, 100.5, 86.9, 76.6, 69.4, 66.8, 56.0, 55.8, 32.3, 31.8, 17.2. HRMS (ESI) calcd for [M]^+^ C_30_H_29_NO_6_: 499.1995, found: 499.2154.

**General procedure for the synthesis of compounds 1 e–g**. To a mixture of acyl halide (0.7 mmol, 1 equiv) and triethylamine (0.15 mmol, 2 equiv) in THF (1.0 mL) was added dropwise a solution of 100 mg of **1** (0.07 mmol, 1 equiv) in THF. The reaction mixture was stirred for 2 h in an ice bath. The reaction was quenched with ammonium chloride (0.5 mL) and the resulting mixture extracted with DCM. The organic layer was dried over MgSO_4_ and concentrated under reduced pressure.

**(6a*R*,12a*S*)‐8,9‐dimethoxy‐2‐(prop‐1‐en‐2‐yl)‐1,2,12,12 a‐tetrahydrochromeno[3,4‐b]furo[2,3‐h]chromen‐6(6aH)‐one O‐acetyl oxime (1 e)**. The residue was purified by flash column chromatography on silica gel (EtOAc/*n*‐hexane=1 : 3) to afford white solid product **1 e** (96 mg, 87 % yield). The obtained acetyl oxime **1 e** data is as follows. ^1^H NMR (500 MHz, CDCl_3_) δ 7.92 (d, *J*=8.6 Hz, 1H), 6.42 (d, *J*=8.7 Hz, 1H), 6.40 (s, 1H), 6.36 (s, 1H), 5.10 (t, *J*=9.0 Hz, 1H), 5.01 (s, 1H), 4.86 (s, 1H), 4.72 (d, *J*=3.1 Hz, 1H), 4.58 (d, *J*=2.5 Hz, 1H), 4.55 (dt, *J*=5.1, 2.4 Hz, 1H), 4.21 (d, *J*=12.0 Hz, 1H), 3.75 (s, 3H), 3.66 (s, 3H), 3.22 (dd, *J*=15.8, 9.8 Hz, 1H), 2.88 (dd, *J*=15.8, 8.2 Hz, 1H), 2.23 (s, 3H), 1.71 (s, 3H). ^13^C NMR (126 MHz, CDCl_3_) δ=168.6, 164.7, 157.2, 153.6, 149.6, 147.6, 143.8, 143.3, 127.1, 113.1, 112.2, 110.9, 106.6, 104.8, 104.4, 100.9, 87.6, 69.3, 66.5, 56.4, 55.8, 33.6, 31.6, 19.8, 17.1. IR (ATR‐FTIR) υ 3073, 2972, 2934, 2855, 2833, 1774, 1622, 1607, 1568, 1513, 1483, 1460, 1351, 1265, 1240, 1214, 1197. HRMS (ESI) calcd for [M+H]^+^ C_25_H_25_NO_7_: 452.1631, found: 452.1707. Melting point: 192.3–194.1 °C.

**(6a*R*,12a*S*)‐8,9‐dimethoxy‐2‐(prop‐1‐en‐2‐yl)‐1,2,12,12 a‐tetrahydrochromeno[3,4‐b]furo[2,3‐h]chromen‐6(6aH)‐one O‐propionyl oxime (1 f)**. The residue was purified by flash column chromatography on silica gel (EtOAc/*n*‐hexane=1 : 3) to afford white solid powder **1 f** (99 mg, 87 % yield). ^1^H NMR (500 MHz, CDCl_3_) δ 7.96 (d, *J*=8.6 Hz, 1H), 6.47 (d, *J*=8.6 Hz, 1H), 6.44 (s, 1H), 6.38 (s, 1H), 5.16 (t, *J*=9.0 Hz, 1H), 5.05 (s, 1H), 4.90 (s, 1H), 4.75 (d, *J*=3.2 Hz, 1H), 4.62 (dd, *J*=12.2, 2.5 Hz, 1H), 4.59–4.56 (m, 1H), 4.27 (d, *J*=12.1 Hz, 1H), 3.80 (s, 3H), 3.69 (s, 3H), 3.27 (dd, *J*=15.7, 9.8 Hz, 1H), 2.91 (dd, *J*=15.8, 8.2 Hz, 1H), 2.53 (q, *J*=5 Hz, 2H), 1.75 (s, 3H), 1.24 (t, *J*=7.5 Hz, 3H). ^13^C NMR (126 MHz, CDCl_3_) δ=172.1, 164.7, 157.2, 153.6, 149.6, 147.5, 143.8, 143.3, 127.2, 113.2, 112.3, 110.7, 106.7, 104.8, 104.5, 100.8, 87.2, 69.3, 66.6, 56.4, 55.9, 33.6, 31.7, 26.6, 17.2, 9.2. IR (ATR‐FTIR) υ 3090, 3064, 2973, 2954, 2936, 2852, 2830, 1779, 1619, 1608, 1583, 1513, 1485, 1457, 1440, 1408, 1378, 1351, 1323, 1265. HRMS (ESI) calcd for [M+H]^+^ C_26_H_28_NO_7_: 466.1866, found: 466.1872. Melting point: 216.6–219.0 °C.

**(6a*R*,12a*S*)‐8,9‐dimethoxy‐2‐(prop‐1‐en‐2‐yl)‐1,2,12,12 a‐tetrahydrochromeno[3,4‐b]furo[2,3‐h]chromen‐6(6aH)‐one O‐butyryl oxime (1 g)**. The residue was purified by flash column chromatography on silica gel (EtOAc/*n*‐hexane=1 : 3) to afford white solid product **1 g** (99 mg, 84 % yield). ^1^H NMR (500 MHz, CDCl_3_) δ 7.96 (d, *J*=8.6 Hz, 1H), 6.46 (d, *J*=8.7 Hz, 1H), 6.43 (s, 1H), 6.38 (s, 1H), 5.15 (t, *J*=8.9 Hz, 1H), 5.04 (s, 1H), 4.89 (s, 1H), 4.74 (d, *J*=3.2 Hz, 1H), 4.61 (dd, *J*=12.1, 2.5 Hz, 1H), 4.58–4.56 (m, 1H), 4.26 (d, *J*=12.1 Hz, 1H), 3.79 (s, 3H), 3.68 (s, 3H), 3.26 (dd, *J*=15.7, 9.7 Hz, 1H), 2.91 (dd, *J*=15.8, 8.2 Hz, 1H), 2.47 (td, *J*=7.3, 1.3 Hz, 2H), 1.79–1.70 (m, 2H), 1.74 (s, 3H), 0.99 (t, *J*=7.4 Hz, 3H). ^13^C NMR (126 MHz, CDCl_3_) δ=171.1, 164.8, 157.3, 153.7, 149.7, 147.7, 143.9, 143.4, 127.3, 113.2, 112.4, 110.8, 106.8, 104.9, 104.6, 100.9, 87.3, 69.4, 66.7, 56.4, 56.0, 35.1, 33.7, 31.8, 18.6, 17.3, 13.9. IR (ATR‐FTIR) υ 3094, 3062, 2961, 2932, 2875, 2847, 2831, 1778, 1620, 1614, 1606, 1514, 1485, 1456, 1442, 1380, 1351, 1338, 1324, 1265. HRMS (ESI) calcd for [M+H]^+^ C_27_H_30_NO_7_: 480.2022, found: 480.2025. Melting point: 238.3–238.5 °C.

**(2*R*,6a*R*,12a*S*)‐8,9‐dimethoxy‐2‐(prop‐1‐en‐2‐yl)‐1,2,6,6 a,12,12 a‐hexahydrochromeno[3,4‐b]furo[2,3‐h]chromen‐6‐ol (2)**.^[15a]^ To a solution of rotenone (1.0 g, 2.55 mmol, 1 equiv) in MeOH (60 mL), NaBH_4_ (0.29 g, 7.66 mmol, 3 equiv) was added. The reaction mixture was stirred at room temperature for 2 h and was quenched with 2 mL of ice‐cold water. The mixture was then transferred to an ice bath and the product was precipitated using generous amount of ice‐cold water. The white precipitate was collected by vacuum filtration to afford compound **2** as white solid powder (972 mg, 96 % yield). The alcohol rotenone derivative matched the reported characterization data. ^1^H NMR (500 MHz, CDCl_3_) δ 7.05 (d, J=8.1 Hz, 1H), 6.70 (s, 1H), 6.46 (s, 1H), 6.45 (s, 1H), 5.21 (t, J=8.8 Hz, 1H), 5.09 (s, 1H), 4.93 (s, 1H), 4.92 (s, 1H), 4.83 (dt, J=11.3, 5.7 Hz, 1H), 4.62 (t, J=10.6 Hz, 1H), 4.23 (dd, J=9.6, 5.1 Hz, 1H), 3.86 (s, 3H), 3.84 (s, 3H), 3.39 (s, 1H), 3.30 (dd, J=15.6, 9.7 Hz, 1H), 2.96 (dd, J=15.6, 8.1 Hz, 1H), 1.83–1.81 (m, 1H, OH) 1.78 (s, 3H). ^13^C NMR (126 MHz, CDCl_3_) δ 161.9, 149.7, 149.4, 149.3 (2 C), 143.9, 130.5, 113.9, 112.9, 112.1, 111.4, 108.9, 102.8, 100.8, 86.7, 69.3, 66.4, 65.1, 56.6, 56.0, 38.2, 32.1, 17.4. IR (ATR‐FTIR) υ 3419, 2768, 1321, 1202, 1150, 1125, 872, 846, 776, 728, cm^−1^. [M−OH]^+^ C_23_H_24_O_6_: 379.1540, found: 379.1562. Melting point: 128–132 °C.

**General procedure for the synthesis of compounds 2 a–d**. To a solution of **2** (1 equiv) in THF, the alkyl halides (1.5 equiv) and potassium *tert*‐butoxide (1.0 M in THF, 1 equiv) were added at 0 °C. After completion of the reaction as determined by TLC, the mixture was quenched with saturated NH_4_Cl solution and was extracted with EtOAc (3x). The organic layer was dried over MgSO_4_, filtered, and concentrated under reduced pressure. The crude residue was purified by column chromatography on silica gel (gradient elution from 0 : 100 to 100:0 EtOAc/*n*‐hexane) to afford the desired product. Alkyl halides used: **2 a**=methyl iodide, **2 b**=ethyl iodide, **2 c**=propyl iodide, **2 d**=benzyl bromide.

**(2*R*,6a*R*,12a*S*)‐6,8,9‐trimethoxy‐2‐(prop‐1‐en‐2‐yl)‐1,2,6,6 a,12,12 a‐hexahydrochromeno[3,4‐b]furo[2,3‐h]chromene (2 a)**. The methoxy derivative **2 a** was obtained as white solid powder (0.39 g, 76 % yield). ^1^H NMR (500 MHz, CDCl_3_) δ 6.99 (d, *J*=8.1 Hz, 1H), 6.79 (s, 1H), 6.44 (s, 1H), 6.42 (d, *J*=8.0 Hz, 1H), 5.19 (t, *J*=8.8 Hz, 1H), 5.09 (s, 1H), 4.91 (s, 1H), 4.78 (d, *J*=5.2 Hz, 1H), 4.55 (t, *J*=10.0 Hz, 1H), 4.42 (s, 1H), 4.23 (dd, *J*=9.8, 4.4 Hz, 1H), 3.84 (s, 6H), 3.42 (s, 1H), 3.30 (dd, *J*=15.7, 9.6 Hz, 1H), 3.17 (s, 3H), 2.95 (dd, *J*=15.7, 8.2 Hz, 1H), 1.78 (s, 3H). ^13^C NMR (126 MHz, CDCl_3_) δ 161.9, 149.8, 149.1, 149.1, 144.0, 143.6, 129.8, 113.6, 113.2, 112.0, 111.4, 110.2, 101.6, 100.4, 86.6, 76.7, 70.1, 65.8, 57.0, 56.6, 55.9, 37.5, 32.1, 17.4. IR (ATR‐FTIR) υ 3039, 2978, 2961, 1612, 1514, 1468, 1442, 1260, 1192, 1130, 1088, 1038. HRMS (ESI) calc'd for [M^+^] C_24_H_26_O_6_: 410.1729, found: 410.1660. Melting point: 114–117 °C.

**6‐Ethoxy‐2‐isopropenyl‐8,9‐dimethoxy‐1,2,6,6 a,12,12 a‐hexahydro‐chromeno[3,4‐b]furo[2,3‐h]chromene (2 b)**. The ethoxy derivative **2 b** was obtained as white solid powder (0.25 g, 46 % yield). ^1^H NMR (500 MHz, CDCl_3_) δ 7.01 (d, *J*=7.9 Hz, 1H), 6.86 (s, 1H), 6.42 (s, 1H), 6.40 (d, *J*=8.1 Hz, 1H), 5.17 (t, *J*=8.9 Hz, 1H), 5.08 (s, 1H), 4.90 (s, 1H), 4.77 (d, *J*=4.7 Hz, 1H), 4.62 (t, *J*=9.7 Hz, 1H), 4.57 (s, 1H), 4.22 (d, *J*=9.8 Hz, 1H), 3.83 (s, 6H), 3.50–3.46 (m, 1H), 3.43 (s, 1H), 3.30 (s, 1H), 3.28 (dd, *J*=19.0, 12.7 Hz, 1H), 2.94 (dd, *J*=15.6, 8.1 Hz, 1H), 1.78 (s, 3H), 1.02 (t, *J*=6.7 Hz, 3H). ^13^C NMR (126 MHz, CDCl_3_) δ 161.6, 149.8, 149.1, 149.0, 144.0, 143.4, 129.1, 114.5, 113.0, 112.0, 111.7, 110.3, 101.8, 100.3, 86.6, 75.1, 70.2, 66.0, 64.9, 56.6, 55.9, 37.0, 32.1, 17.4, 15.4. IR (ATR‐FTIR) υ 3103, 2999, 2909, 1620, 1512, 1477, 1458, 1441, 1364, 1342, 1306, 1217, 1194, 1180, 1165, 1101, 1082. HRMS (ESI) calc'd for [M+H]^+^ C_25_H_29_O_6_: 425.1970, found: 425.2124. Melting point: 155–158 °C.

**2‐Isopropenyl‐8,9‐dimethoxy‐6‐propoxy‐1,2,6,6 a,12,12 a‐hexahydro‐chromeno[3,4‐b]furo[2,3‐h]chromene (2 c)**. The propoxy derivative **2 c** was obtained as white solid powder in 87 % yield. ^1^H NMR (500 MHz, CDCl_3_) δ 7.00 (d, *J*=7.8 Hz, 1H), 6.85 (s, 1H), 6.42 (s, 1H), 6.39 (s, 1H), 5.18 (t, *J*=8.8 Hz, 1H), 5.08 (s, 1H), 4.90 (s, 1H), 4.88–4.70 (m, 1H), 4.61 (d, *J*=9.7 Hz, 1H), 4.54 (s, 1H), 4.22 (d, *J*=9.5 Hz, 1H), 3.82 (s, 6H), 3.47–3.37 (m, 2H), 3.28 (dd, *J*=15.5, 9.9 Hz, 1H), 3.18 (d, *J*=6.9 Hz, 1H), 2.94 (dd, *J*=15.6, 8.1 Hz, 1H), 1.77 (s, 3H), 1.45–1.36 (m, 2H), 0.70 (t, *J*=7.2 Hz, 3H). ^13^C NMR (126 MHz, CDCl_3_) δ 161.5, 149.7, 149.1, 148.9, 144.0, 143.3, 129.0, 114.3, 112.9, 111.9, 111.6, 110.3, 101.6, 100.2, 86.5, 75.2, 71.3, 70.1, 65.9, 56.5, 55.8, 36.9, 32.1, 23.0, 17.3, 10.7. IR (ATR‐FTIR) υ 3098, 2997, 2913, 1618, 1607, 1510, 1477, 1460, 1368, 1342, 1329, 1304, 1260, 1215. HRMS (ESI) calc'd for [M+Na]^+^ C_26_H_30_O_6_Na: 461.1940, found: 461.2330. Melting point: 124–127 °C.

**(2*R*,6a*R*,12a*S*)‐6‐(benzyloxy)‐8,9‐dimethoxy‐2‐(prop‐1‐en‐2‐yl)‐1,2,6,6 a,12,12 a‐hexahydrochromeno[3,4‐b]furo[2,3‐h]chromene (2 d)**. The benzyl derivative **2 d** was obtained as while solid powder (0.57 g, 94 % yield). ^1^H NMR (500 MHz, CDCl_3_) δ 7.24 (s, 3H), 7.01 (d, *J*=5.3 Hz, 2H), 6.94 (d, *J*=7.9 Hz, 1H), 6.63 (s, 1H), 6.49 (s, 1H), 6.43 (d, *J*=7.9 Hz, 1H), 5.21 (t, *J*=8.8 Hz, 1H), 5.10 (s, 1H), 4.92 (s, 1H), 4.83 (m, 1H), 4.67 (t, *J*=10.1 Hz, 1H), 4.58 (s, 1H), 4.41 (dd, *J*=87.2, 12.5 Hz, 2H), 4.30–4.24 (m, 1H), 3.88 (s, 3H), 3.75 (s, 3H), 3.41 (m, 1H), 3.32 (dd, *J*=15.5, 9.8 Hz, 1H), 2.97 (dd, *J*=15.5, 8.2 Hz, 1H), 1.80 (s, 3H). ^13^C NMR (126 MHz, CDCl_3_) δ=162.0, 150.1, 149.1, 149.0, 144.0, 143.5, 138.1, 129.7, 128.3, 127.8, 127.5, 113.7, 113.3, 112.0, 111.3, 110.2, 101.6, 100.5, 86.7, 72.7, 70.0, 69.8, 65.9, 56.4, 56.0, 37.6, 32.1, 17.4. IR (ATR‐FTIR) υ 3057, 3036, 2924, 2858, 1620, 1609, 1508, 1477, 1454, 1412, 1369, 1346, 1308, 1273, 1215, 1192, 1177, 1161, 1099, 1080. HRMS (ESI) calc'd for [M+Na]^+^ C_30_H_30_O_6_Na: 509.1940, found: 509.2410. Melting point: 154–156 °C.

**(2*R*,6a*R*,12a*S*)‐8,9‐dimethoxy‐2‐(prop‐1‐en‐2‐yl)‐6‐((tetrahydro‐2H‐pyran‐2‐yl)oxy)‐1,2,6,6 a,12,12 a‐hexahydrochromeno[3,4‐b]furo[2,3‐h]chromene (2 e)**. To a mixture of **2** (0.10 g, 0.25 mmol, 1 equiv) and DHP (41 mg, 0.5 mmol, 2 equiv) in CH_2_Cl_2_ (1.0 mL) was added pyridinium *p*‐toluenesulfonate (15.9 mg, 0.06 mmol, 0.25 equiv). The reaction mixture was stirred for 1 h and was quenched with water (0.5 mL). The resulting mixture was extracted with EtOAc. The organic layer was dried over MgSO_4_ and was concentrated under reduced pressure. The residue was purified by column chromatography on silica gel (gradient elution from 0 : 100 to 100:0 EtOAc/*n*‐hexane) and recrystallized in hexane to afford **2 e** as white solid (7.9 mg, 63 % yield) (diastereomeric mixture). ^1^H NMR (500 MHz, CDCl_3_) δ 7.12 (d, *J*=7.5 Hz, 1H), 6.97 (d, *J*=7.4 Hz, 1H), 6.85 (s, 1H), 6.74 (s, 1H), 6.56–6.26 (m, 4H), 5.19 (s, 2H), 5.08 (s, 2H), 4.90 (s, 4H), 4.82 (d, *J*=13.0 Hz, 2H), 4.75 (s, 1H), 4.63 (dt, *J*=39.9, 10.0 Hz, 3H), 4.34 (s, 1H), 4.23 (dd, *J*=16.9, 7.2 Hz, 2H), 3.82 (d, *J*=9.0 Hz, 13H), 3.71 (t, *J*=10.6 Hz, 1H), 3.42 (s, 4H), 3.32–3.25 (m, 3H), 3.11 (d, *J*=9.4 Hz, 1H), 2.95 (dd, *J*=14.8, 7.1 Hz, 2H), 1.77 (s, 6H), 1.54–1.20 (m, 14H). ^13^C NMR (126 MHz, CDCl_3_) δ 161.95, 161.46, 150.08, 149.65, 149.08, 144.07, 144.00, 143.46, 133.09, 130.06, 129.77, 128.44, 113.23, 112.49, 111.99, 111.97, 111.75, 102.46, 101.52, 100.41, 100.32, 86.62, 86.61, 77.16, 72.49, 69.93, 69.59, 69.31, 65.88, 64.11, 61.73, 6060, 56.67, 56.67, 56.62, 56.02, 55.99, 37.52, 32.15, 30.44, 30.36, 29.81, 25.58, 25.54, 18.75, 18.23, 17.36, 17.33, 14.25. FTIR υ 3082, 3028, 2992, 2941, 2868, 1616, 1504, 1462, 1440, 1269, 1231, 1211 cm^−1^. HRMS (ESI) calcd for [M+Na]^+^ C_28_H_32_O_7_: 503.2046, found: 503.2037. Melting point: 146.3–147.9 °C.

**(2*R*,6a*R*,12a*S*)‐8,9‐dimethoxy‐2‐(prop‐1‐en‐2‐yl)‐1,2,6,6 a,12,12 a‐hexahydrochromeno[3,4‐b]furo[2,3‐h]chromen‐6‐yl carbamate (2 f)**. To a solution of **2** (0.10 mg, 0.25 mmol, 1 equiv) in CH_2_Cl_2_ (3.0 mL), trichloroacetyl isocyanate (60.1 μL, 0.50 mmol, 2 equiv) was added at 0 °C. The reaction mixture was stirred for 30 min, and then methanol (0.5 mL, 1.26 mmol, 10 equiv) and water (0.5 mL) were added. The resulting mixture was then stirred for 2.5 h at room temperature and was extracted with EtOAc. The organic layer was washed with brine, dried over MgSO_4_, and concentrated under reduced pressure. The residue was purified by column chromatography on silica gel (gradient elution from 0 : 100 to 100:0 EtOAc/n‐hexane) to give the desired product. The amide derivative **2 f** appears as a yellowish white solid (68.7 mg, 62 % yield). ^1^H NMR (500 MHz, CDCl_3_) δ 7.00 (s, 1H), 6.86 (d, *J*=8.0 Hz, 1H), 6.61 (s, 1H), 6.41 (s, 2H), 6.40 (s, 1H), 5.33–5.21 (m, 1H), 5.18 (t, *J*=8.8 Hz, 1H), 5.09 (s, 1H), 4.92 (s, 1H), 4.57 (dd, J=9.7, 5.4 Hz, 1H), 4.12 (t, *J*=10.4 Hz, 1H), 3.90 (s, 3H), 3.85 (s, 3H), 3.25 (dd, *J*=15.6, 9.5 Hz, 1H), 2.97 (dd, *J*=15.6, 7.9 Hz, 1H), 1.79 (s, 3H). ^13^C NMR (126 MHz, CDCl_3_) δ 161.3, 150.3, 149.5, 149.2, 144.7, 143.8, 126.8, 123.3, 116.9, 112.9, 112.3, 112.1, 110.9, 105.1, 102.9, 101.0, 86.7, 71.1, 68.0, 56.4, 56.1, 31.8, 17.4. IR (ATR‐FTIR) υ 3364, 3317, 3240, 3178, 2924, 1690, 1612, 1512, 1466, 1381, 1350, 1265, 1219, 1196, 1142, 1088, 1018 cm^−1^. Melting point: 76–79 °C.

**General procedure for the synthesis of compounds 2 g and 2 h**. To a solution of **2** (1 equiv) and DMAP (catalytic amount) in CH_2_Cl_2_ (1.0 mL), Et_3_N (1 equiv) and anhydride (8 equiv) were added at 0 °C. The reaction mixture was stirred at ambient temperature and the reaction was monitored by TLC. The reaction was then quenched with saturated NH_4_Cl solution. The mixture was extracted with CH_2_Cl_2_ (3x). The organic layer was washed with brine, dried over MgSO_4_, and concentrated under reduced pressure. The residue was purified by column chromatography on silica gel (gradient elution from 0 : 100 to 100:0 EtOAc/*n*‐hexane) to afford the desired product. Anhydrides used: **2 g**=acetic anhydride and **2 h**=succinic anhydride

**(2*R*,6a*R*,12a*S*)‐8,9‐dimethoxy‐2‐(prop‐1‐en‐2‐yl)‐1,2,6,6 a,12,12 a‐hexahydrochromeno[3,4‐b]furo[2,3‐h]chromen‐6‐yl acetate (2 g)**. The acetyl derivative **2 g** was obtained as a white solid powder (77 mg, 69 % yield). ^1^H NMR (500 MHz, CDCl_3_) δ 7.09 (d, *J*=8.2 Hz, 1H), 6.65 (s, 1H), 6.46–6.39 (m, 2H), 6.30 (d, *J*=4.4 Hz, 1H), 5.22 (t, *J*=8.9 Hz, 1H), 5.09 (s, 1H), 4.94–4.91 (m, 1H), 4.87 (dt, *J*=11.3, 5.6 Hz, 1H), 4.48 (dd, *J*=11.3, 10.1 Hz, 1H), 4.26 (ddd, *J*=10.0, 5.2, 1.3 Hz, 1H), 3.84 (s, 6H), 3.54 (t, *J*=5.3 Hz, 1H), 3.30 (dd, *J*=15.7, 9.7 Hz, 1H), 2.96 (dd, *J*=15.7, 8.1 Hz, 1H), 1.78 (s, 3H), 1.74 (s, 3H). ^13^C NMR (126 MHz, CDCl_3_) δ 170.1, 162.2, 149.9, 149.3, 148.6, 143.8, 143.5, 131.1, 112.8, 112.2, 111.7, 111.1, 108.6, 103.0, 100.1, 86.8, 69.0, 66.7, 64.6, 56.4, 55.9, 36.6, 32.0, 21.0, 17.3. IR (ATR‐FTIR) υ 3086, 2986, 2955, 2909, 2855, 2832, 1728, 1620, 1605, 1512, 1481, 1443, 1412, 1366, 1327, 1265, 1226. HRMS (ESI) calc'd for [M^+^] C_25_H_26_O_7_: 438.1679, found: 438.1616. Melting point: 139–142 °C.

**4‐(((2*R*,6a*R*,12a*S*)‐8,9‐dimethoxy‐2‐(prop‐1‐en‐2‐yl)‐1,2,6,6 a,12,12 a‐hexahydrochromeno[3,4‐b]furo[2,3‐h]chromen‐6‐yl)oxy)‐4‐oxobutanoic acid (2 h)**. The succinic anhydride derivative **2 h** was obtained as white solid powder (0.50 mg, 79 % yield). ^1^H NMR (500 MHz, CDCl_3_) δ 7.08 (d, *J*=8.1 Hz, 1H), 6.63 (s, 1H), 6.43 (d, *J*=8.1 Hz, 1H), 6.40 (s, 1H), 6.31 (s, 1H), 5.21 (t, *J*=8.9 Hz, 1H), 5.08 (s, 1H), 4.91 (s, 1H), 4.86 (dd, *J*=11.4, 5.2 Hz, 1H), 4.43 (t, *J*=10.7 Hz, 1H), 4.28–4.24 (m, 1H), 3.82 (s, 6H), 3.77 (d, *J*=9.7 Hz, 1H), 3.55 (s, 1H), 3.30 (dd, *J*=15.4, 9.7 Hz, 1H), 2.95 (dd, *J*=15.5, 7.8 Hz, 1H), 2.42–2.13 (m, 4H), 1.78 (s, 3H). ^13^C NMR (126 MHz, DMSO‐*d_6_*) δ 173.6, 171.8, 161.7, 150.1, 149.4, 148.7, 144.2, 143.3, 130.2, 113.4, 112.6, 112.1, 111.9, 109.4, 102.6, 100.7, 86.2, 69.2, 67.8, 64.8, 56.5, 55.8, 35.4, 31.7, 29.3, 28.9, 17.4. FTIR υ 3080, 2956, 2933, 1784, 1730, 1714, 1620, 1514, 1481, 1467, 1411, 1352, 1261, 1219. HRMS (ESI) calc'd for [M+Na]^+^ C_27_H_28_O_9_Na: 519.1631, found: 519.2330. Melting point: 83.5–84.7 °C.

**(2*R*,12a*S*)‐8,9‐dimethoxy‐2‐(prop‐1‐en‐2‐yl)‐1,2,12,12 a‐tetrahydrochromeno[3,4‐b]furo[2,3‐h]chromene (2 i)**. A solution of **2** (0.20 mg, 0.51 mmol, 1 equiv) in acetic acid (5 mL) was stirred for 2 h at 100 °C and was treated with water (2.0 mL). The mixture was extracted with diethyl ether (3x). The organic layer was washed with brine, dried over MgSO_4_, and concentrated under reduced pressure. The residue was purified by column chromatography on silica gel (gradient elution from 0 : 100 to 100:0 EtOAc/*n*‐hexane) to afford **2 i** as a yellow solid powder in 100 % yield. ^1^H NMR (500 MHz, CDCl_3_) δ 7.69 (d, *J*=8.5 Hz, 1H), 6.62 (s, 1H), 6.45 (s, 1H), 6.43 (s, 1H), 5.15 (t, *J*=8.9 Hz, 1H), 5.06 (s, 1H), 4.90 (s, 1H), 4.87 (d, *J*=3.1 Hz, 1H), 4.61 (dd, *J*=12.0, 1.9 Hz, 1H), 4.53 (s, 1H), 4.26 (d, *J*=12.0 Hz, 1H), 3.80 (s, 3H), 3.74 (s, 3H), 3.29 (dd, *J*=15.7, 9.8 Hz, 1H), 2.93 (dd, *J*=15.7, 8.2 Hz, 1H), 1.76 (s, 3H). ^13^C NMR (126 MHz, CDCl_3_) δ=161.3, 150.4, 149.5, 149.3, 144.8, 143.9, 126.9, 123.3, 116.9, 112.9, 112.2, 112.1, 110.9, 105.3, 102.9, 101.1, 86.7, 71.1, 68.0, 56.5, 56.1, 31.8, 17.4. IR (ATR‐FTIR) υ 3086, 3032, 2970, 2916, 2870, 1612, 1504, 1458, 1381, 1342, 1273, 1188, 1149, 1088, 1049 cm^−1^. HRMS (ESI) calc'd for [M^+^] C_23_H_22_O_5:_ 378.1467, found: 378.1529. Melting point: 164–168 °C.

### MTT Assay

The MTT cytotoxicity assay performed in this study was adapted from literature.^[29]^ Cells were seeded at 4 or 6×10^4^ cells/mL (depending on the cell culture used) in sterile 96‐well microtiter plates. The plates were incubated overnight at 37 °C and 5 % CO_2_. Eight two‐fold dilutions of the sample were used as treatments starting from 100 μg/mL down to 0.78 μg/mL. Doxorubicin served as positive control while dimethyl sulfoxide (DMSO) served as negative control. Following incubation, cells were treated with each compound dilution. The treated cells were again incubated for 72 h at 37 °C and 5 % CO_2_. After incubation, the media was removed and 3‐(4,5‐dimethylthiazol‐2‐yl)‐2,5‐diphenyltetrazolium bromide (MTT) dye at 5 mg/mL PBS was added. The cells were again incubated at 37 °C and 5 % CO_2_ for 4 h. After which, DMSO is used to dissolve the formazan crystals formed by the reduction of the dye by the live cells. Absorbance was read at 570 nm. The inhibition concentration 50 (IC_50_) was computed using GraphPad Prism 6. GraphPad Prism 6 computes for the IC_50_ of the sample by employing non‐linear regression curve fit on the computed percent inhibition per concentration of the sample. Samples with IC_50_ values less than 10 μM are considered active.

## Supporting Information

Supporting Information contains ^1^H and ^13^C NMR spectra for all synthesized compounds, MTT assay data, and the SwissADME predictions on the pharmacokinetics and drug‐likeness properties of the rotenone derivatives.

## Conflict of interest

The authors declare no conflict of interest.

1

## Supporting information

As a service to our authors and readers, this journal provides supporting information supplied by the authors. Such materials are peer reviewed and may be re‐organized for online delivery, but are not copy‐edited or typeset. Technical support issues arising from supporting information (other than missing files) should be addressed to the authors.

Supporting Information

## Data Availability

The data that support the findings of this study are available in the supplementary material of this article.
