# Supplementary material for: Antiproliferative Activities and SwissADME Predictions of Physicochemical Properties of Carbonyl Group‐Modified Rotenone Analogues
Source: ChemistryOpen. 2023 Aug 17;13(1):e202300087. doi: 10.1002/open.202300087 (PMC10784630; doi:10.1002/open.202300087)
Supplement: Supplementary file 1 — Supporting Information [file OPEN-13-e202300087-s001.pdf]

ChemistryOpen

Supporting Information

Antiproliferative Activities and SwissADME Predictions of Physicochemical Properties of Carbonyl Group-Modified Rotenone Analogues

Rajelle D. Hernandez, Frances Abygail F. Genio, Jannelle R. Casanova, Marlon T. Conato, and Monissa C. Paderes*

I. ^{1}H and ^{13}C NMR spectra ……………………………………………………….2

II. MTT assay data …..………………………………………………………….....19

III. SwissADME predictions on pharmacokinetics ……………………………22

IV. Drug-likeness properties of the rotenone derivatives …………………….23

I. ^{1}H and ^{13}C NMR spectra

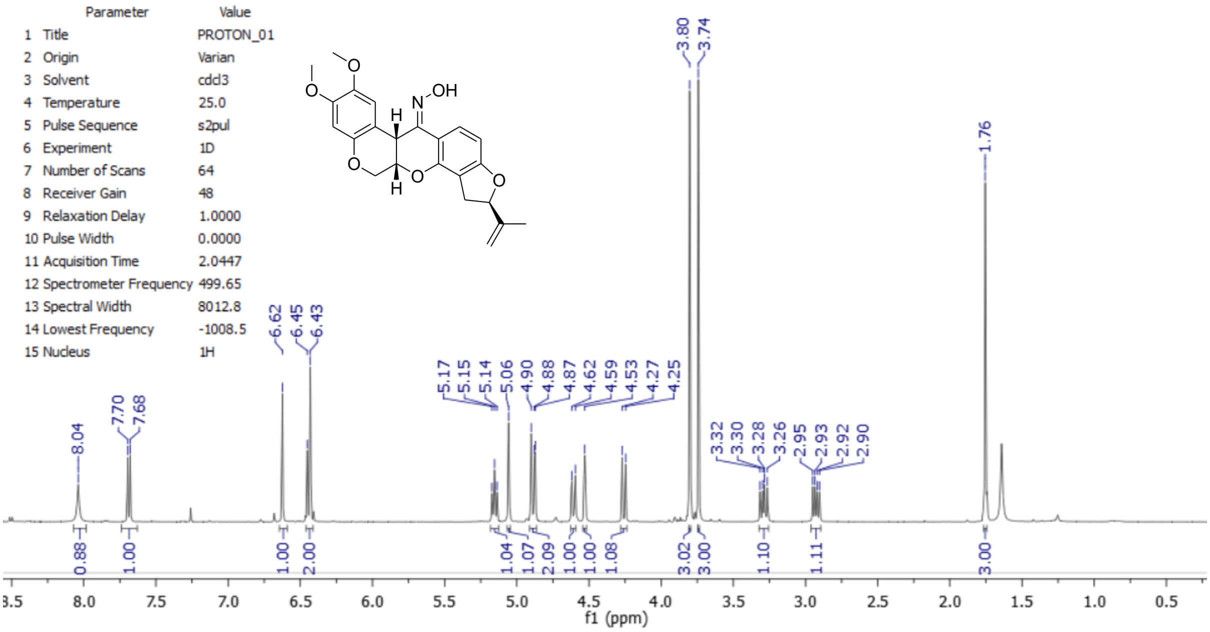

Figure S1. ^{1}H NMR spectra of compound **1**.

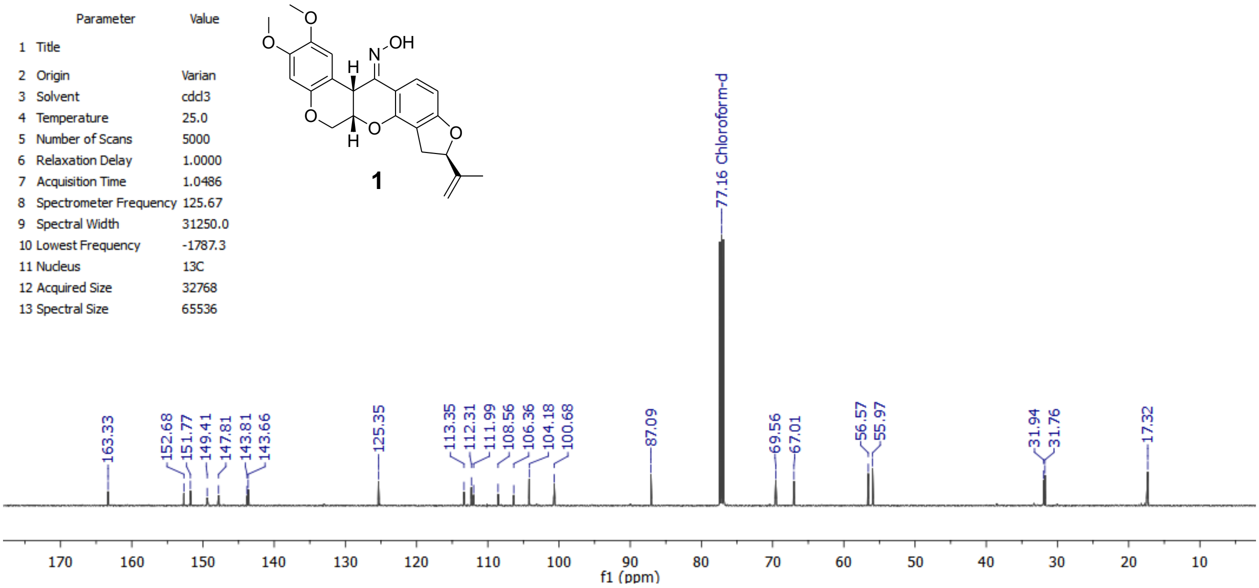

Figure S2. ^{13}C NMR spectra of compound **1**.

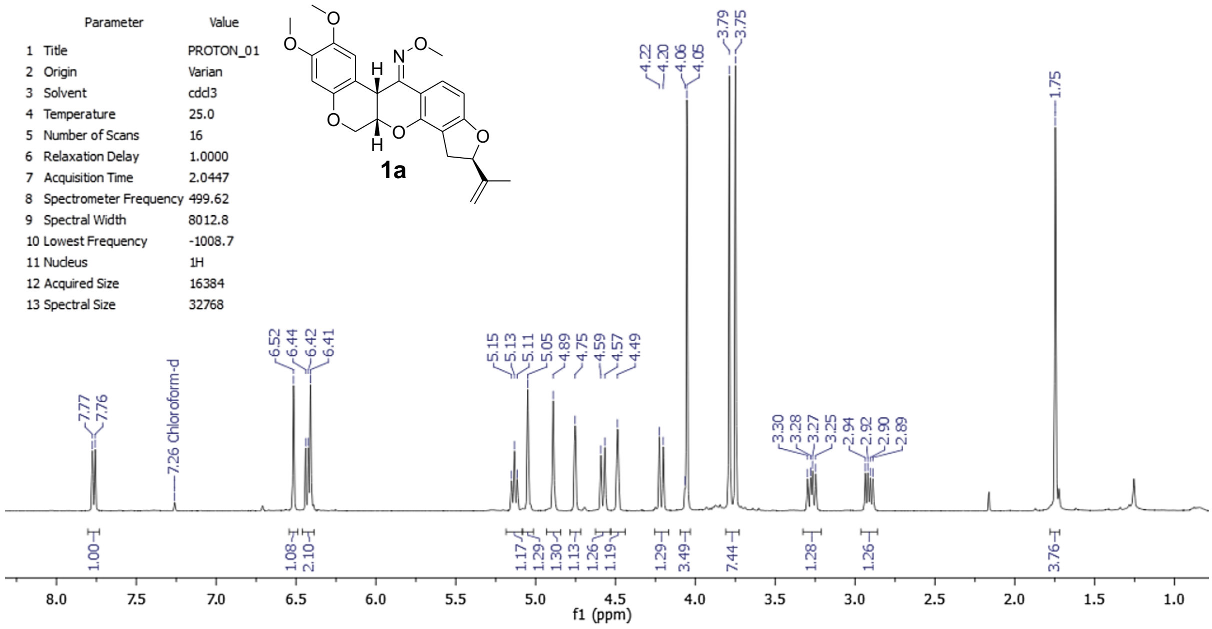

Figure S3. ^{1}H NMR spectra of compound **1a**.

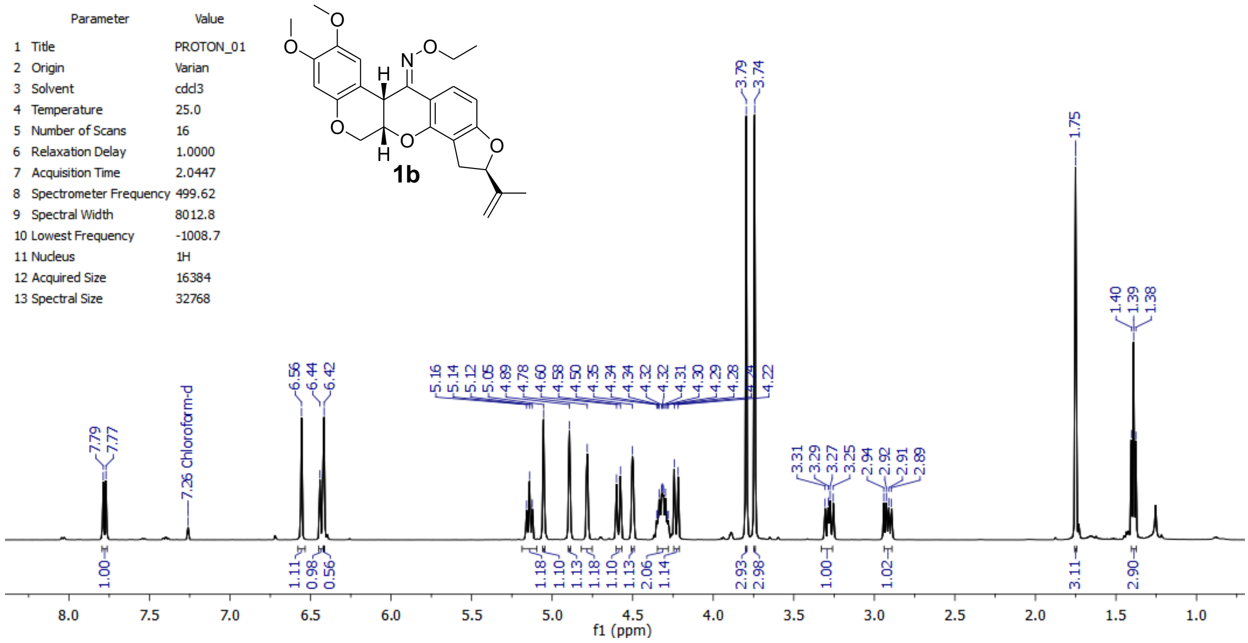

Figure S4. ^{1}H NMR spectra of compound **1b**.

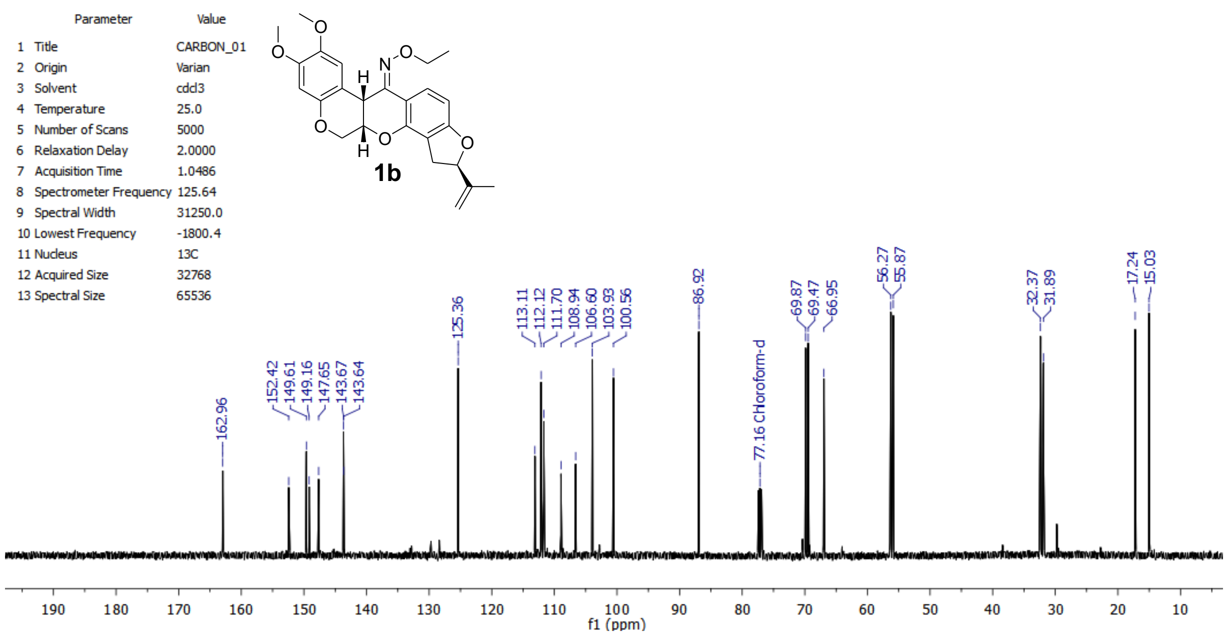

Figure S5. ^{13}C NMR spectra of compound **1b**.

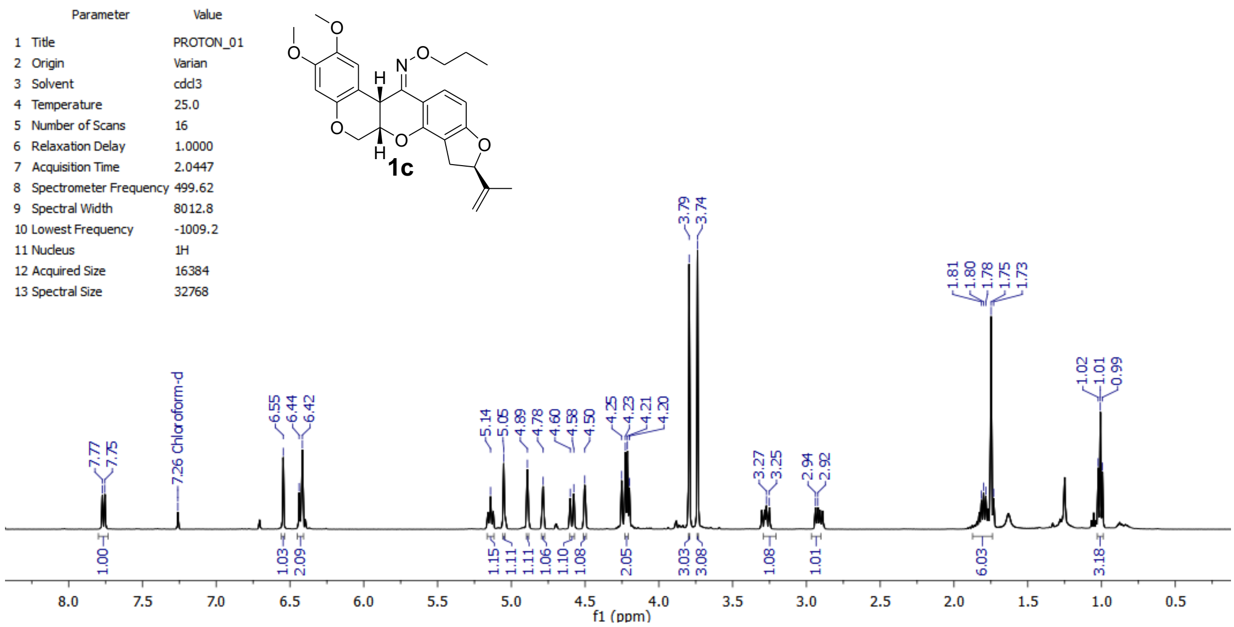

Figure S6. ^{1}H NMR spectra of compound **1c**.

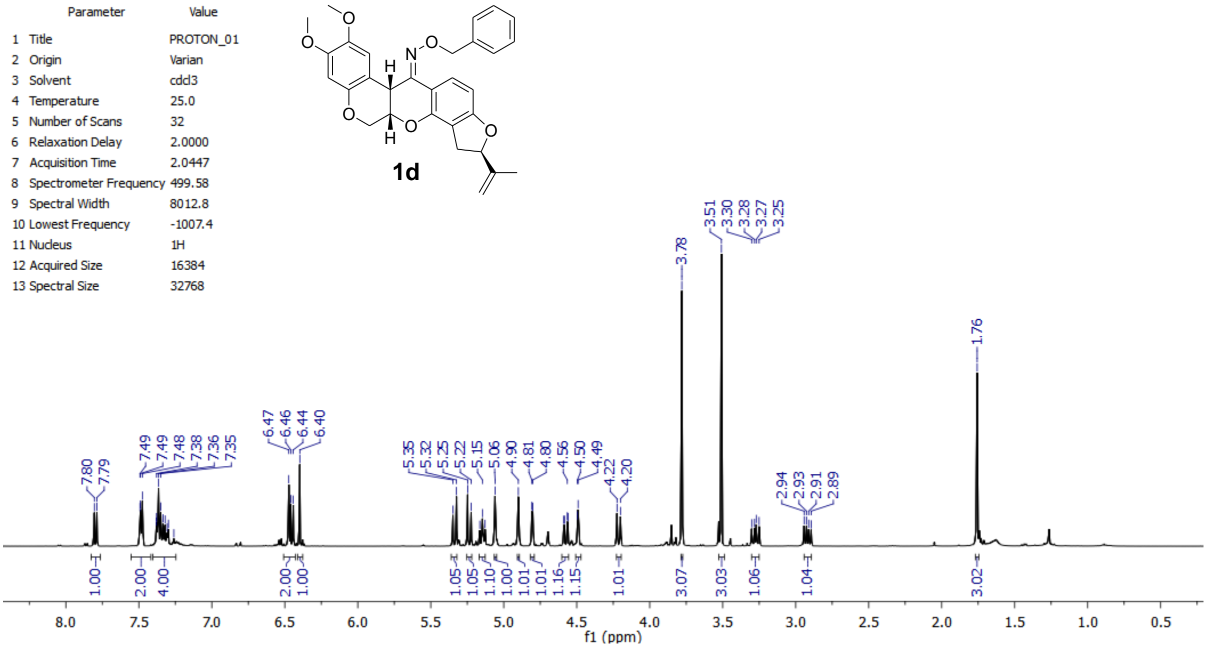

Figure S7. ^{1}H NMR spectra of compound **1d**.

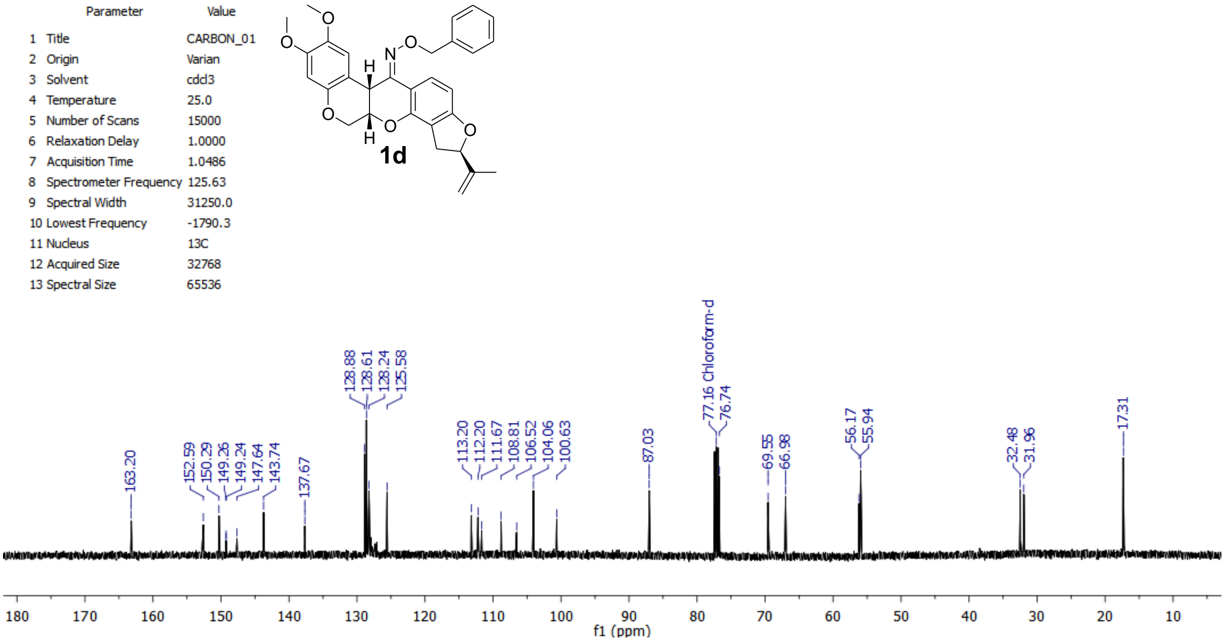

Figure S8. ^{13}C NMR spectra of compound **1d**.

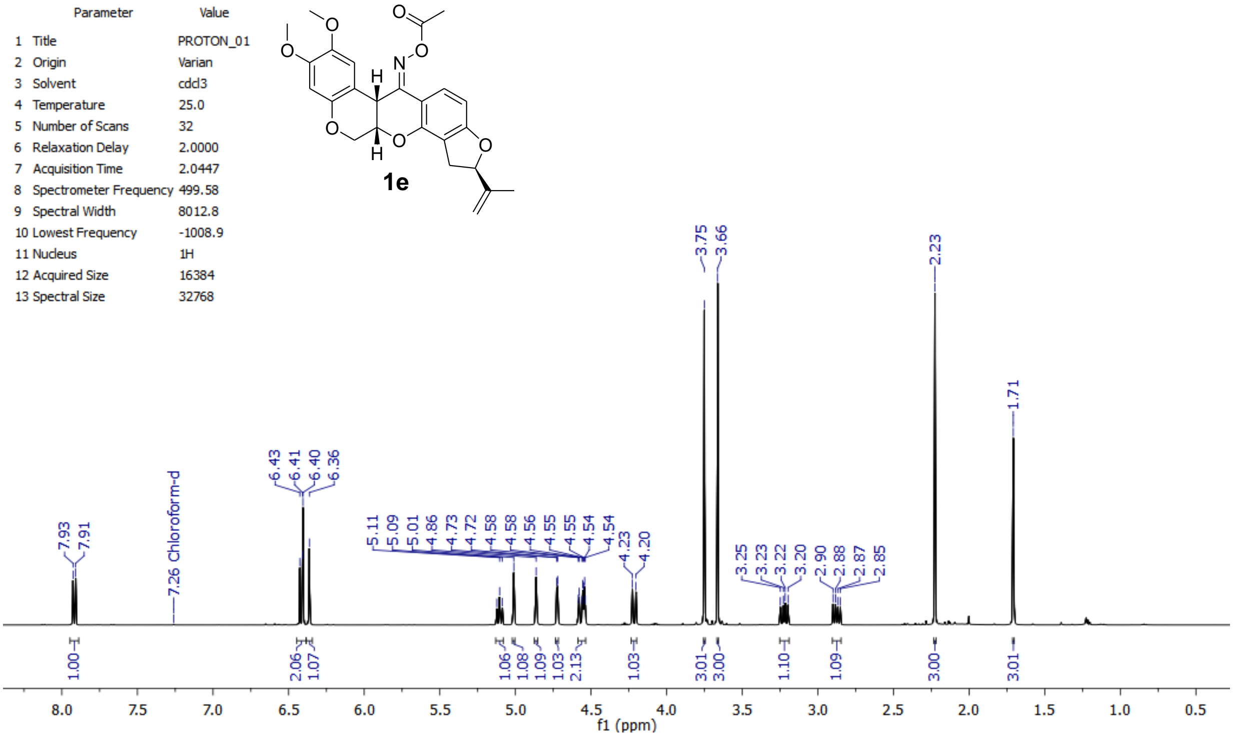

Figure S9. ^{1}H NMR spectra of compound **1e**.

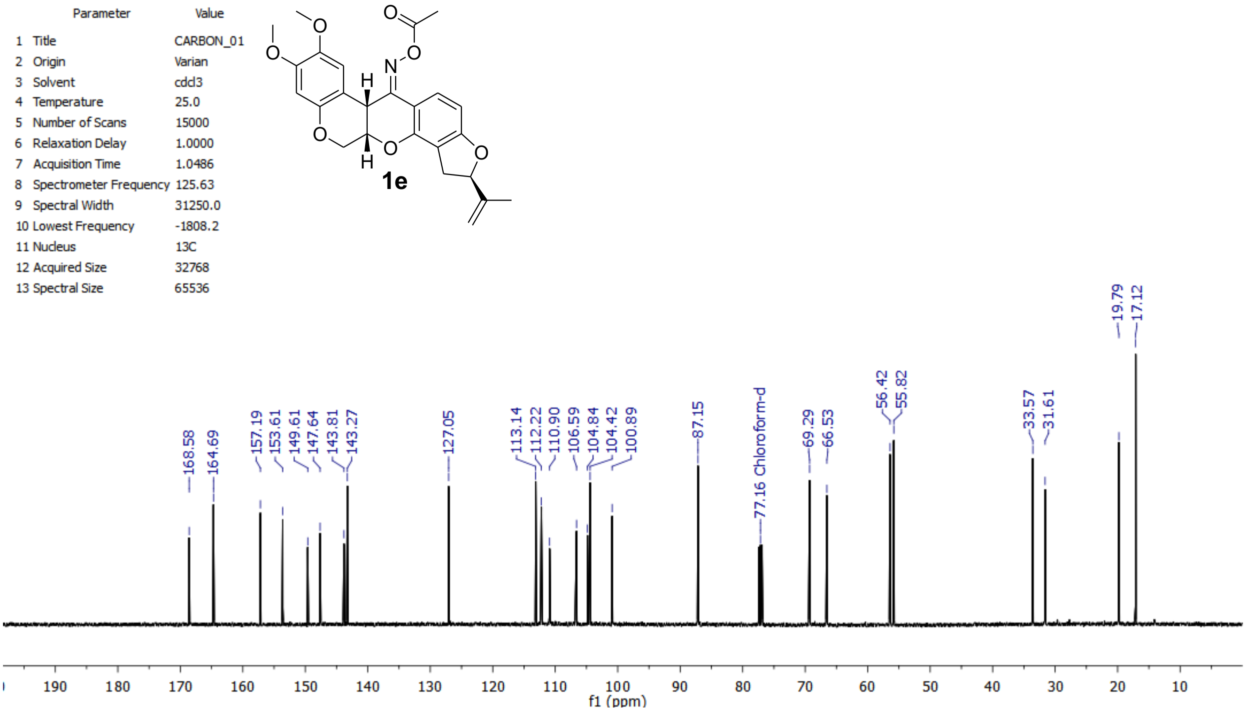

Figure S10. ^{13}C NMR spectra of compound **1e**.

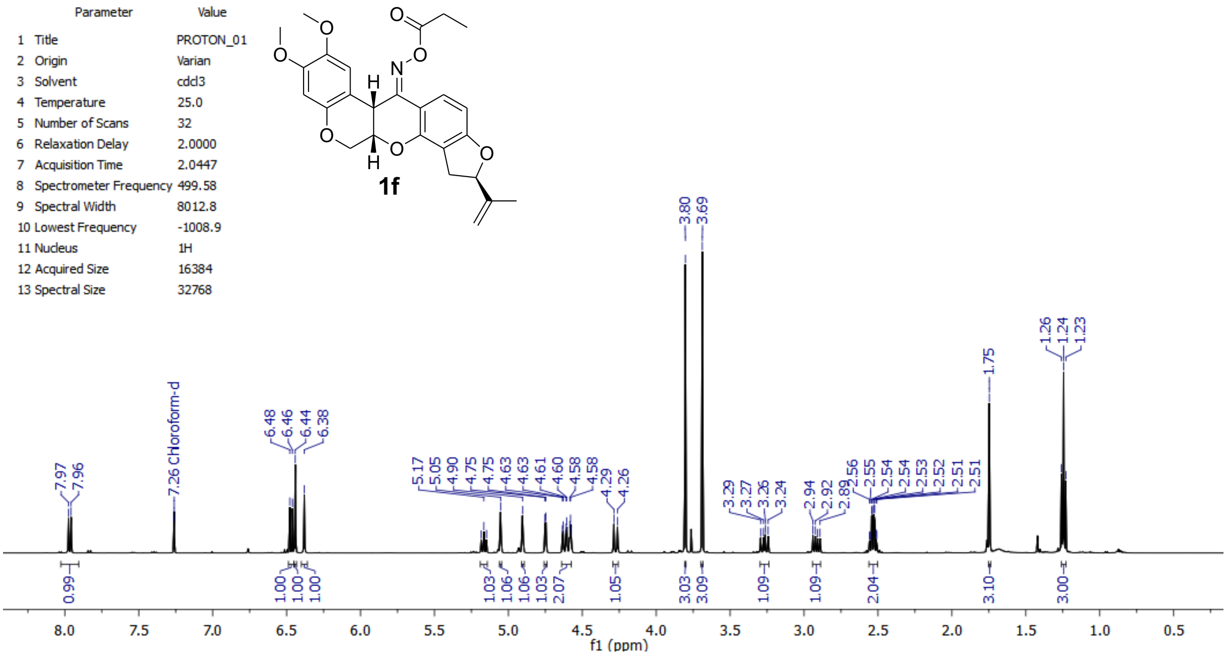

Figure S11. ^{1}H NMR spectra of compound **1f.**

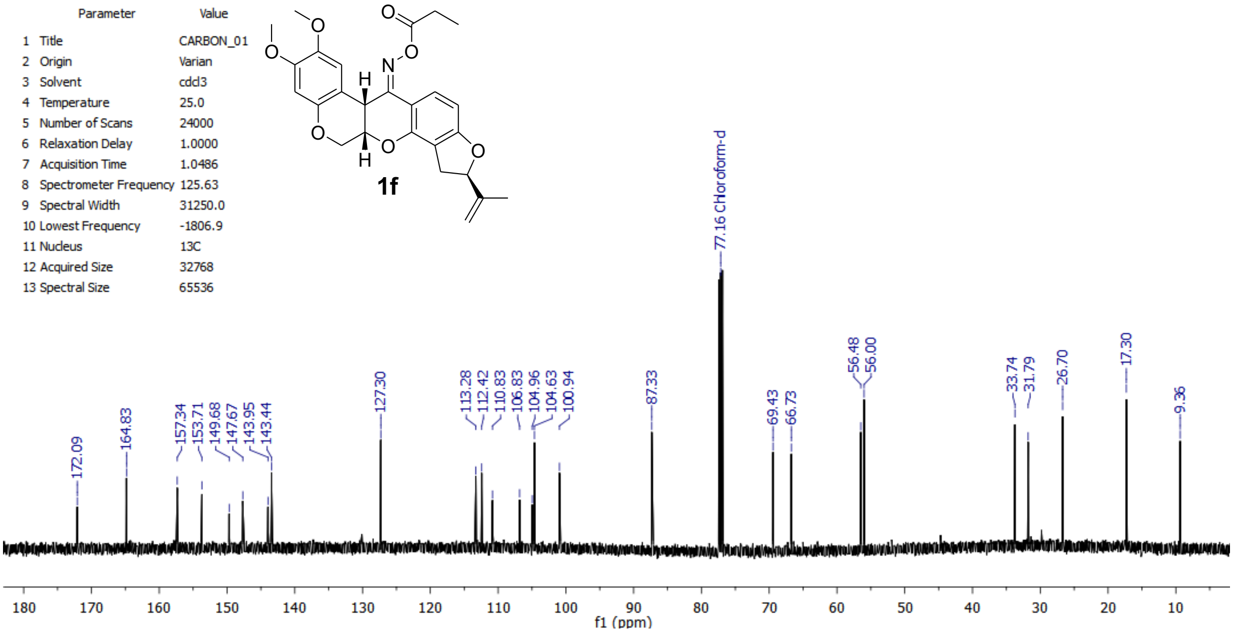

Figure S12. ^{13}C NMR spectra of compound **1f**.

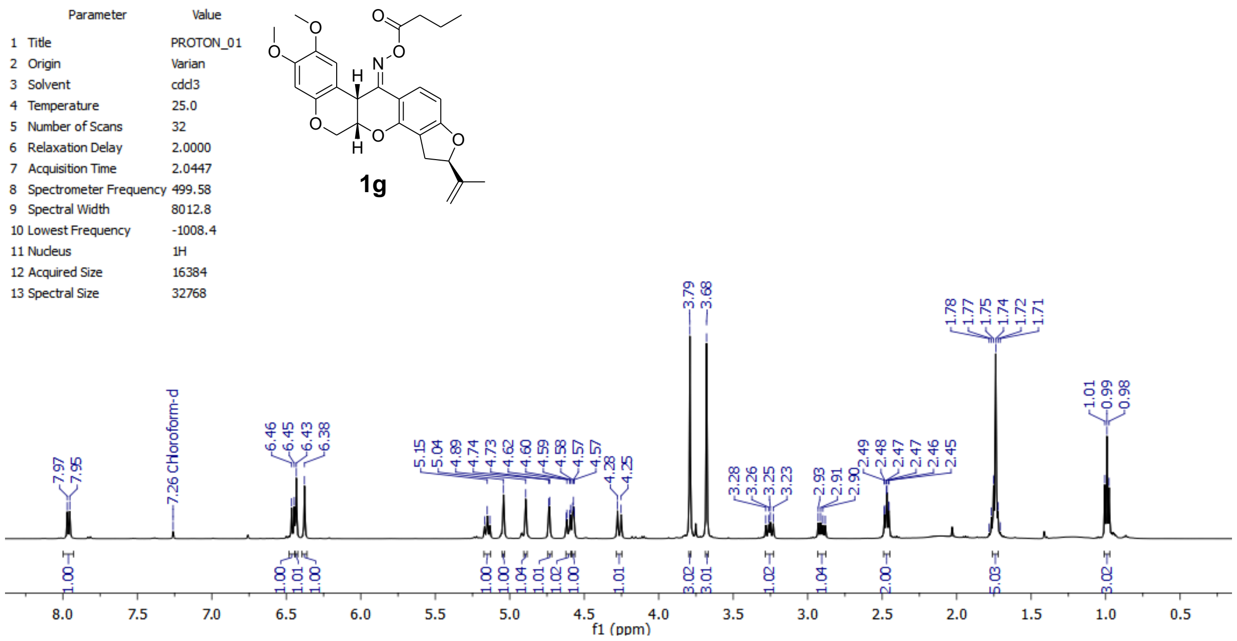

Figure S13. ^{1}H NMR spectra of compound **1g**.

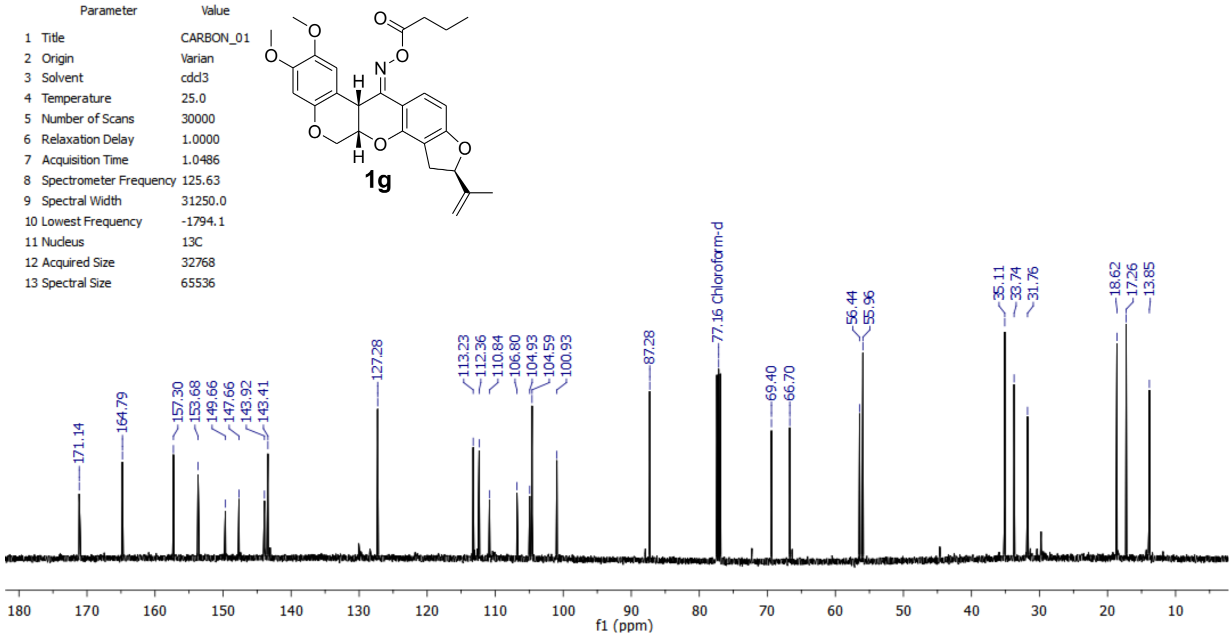

Figure S14. ^{13}C NMR spectra of compound **1g**.

Parameter	Value
1 Title	
2 Solvent	
3 Temperature	25.0
4 Number of Scans	32
5 Relaxation Delay	2.0000
6 Acquisition Time	2.0477
7 Spectrometer Frequency	499.64
8 Spectral Width	8012.8
9 Lowest Frequency	-1008.1
10 Nucleus	1H
11 Acquired Size	
12 Spectral Size	32768

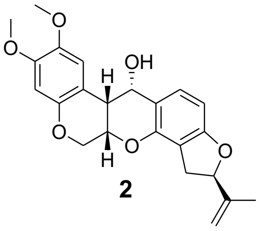

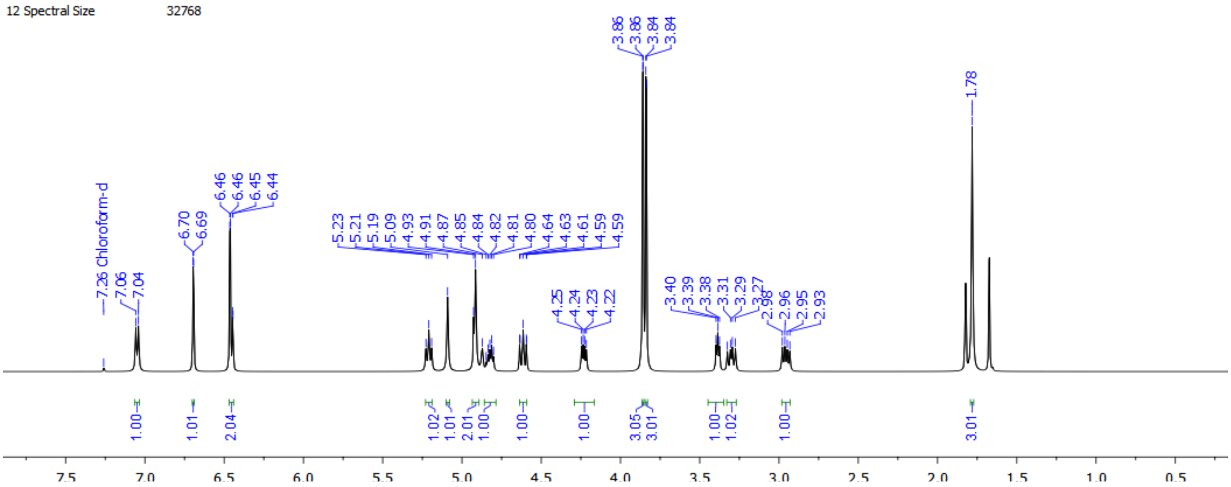

Figure S15. ^{1}H NMR spectra of compound **2**.

Parameter	Value
1 Title	CARBON_01
2 Origin	Varian
3 Solvent	cdcl3
4 Temperature	25.0
5 Number of Scans	4700
6 Relaxation Delay	1.0000
7 Acquisition Time	1.0486
8 Spectrometer Frequency	125.65
9 Spectral Width	31250.0
10 Lowest Frequency	-1791.6
11 Nucleus	13C
12 Acquired Size	32768
13 Spectral Size	65536

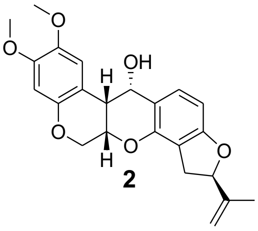

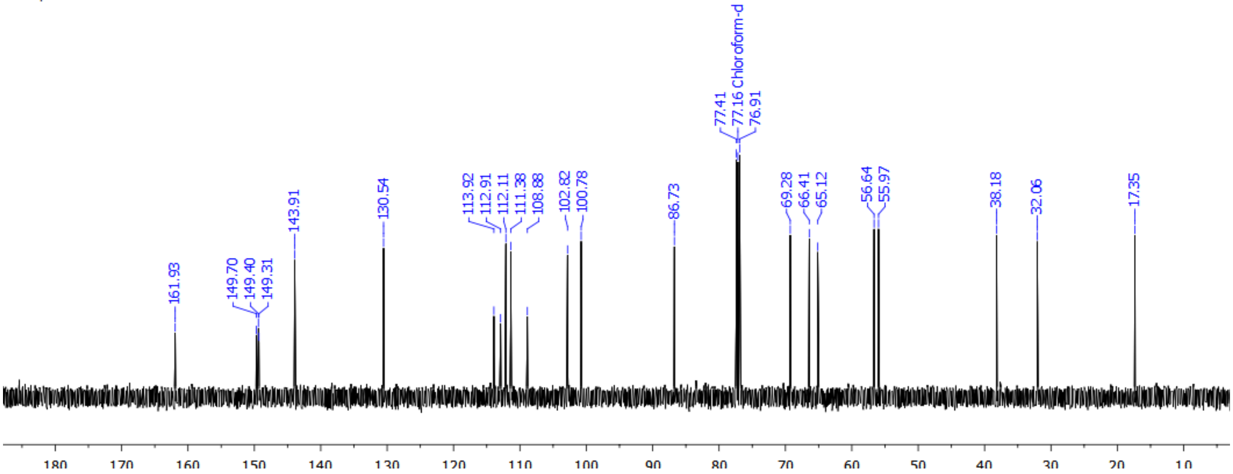

Figure S15. ^{13}C NMR spectra of compound **2**.

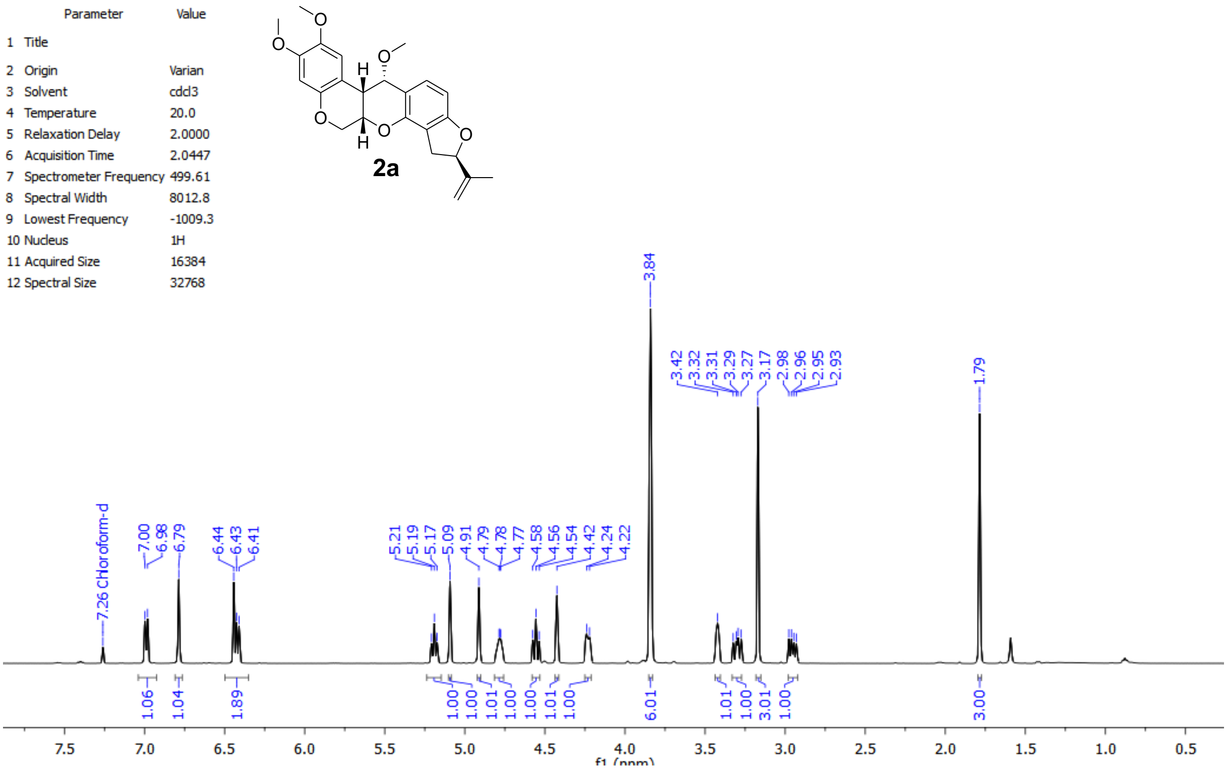

Figure S16. ^{1}H NMR spectra of compound **2a**.

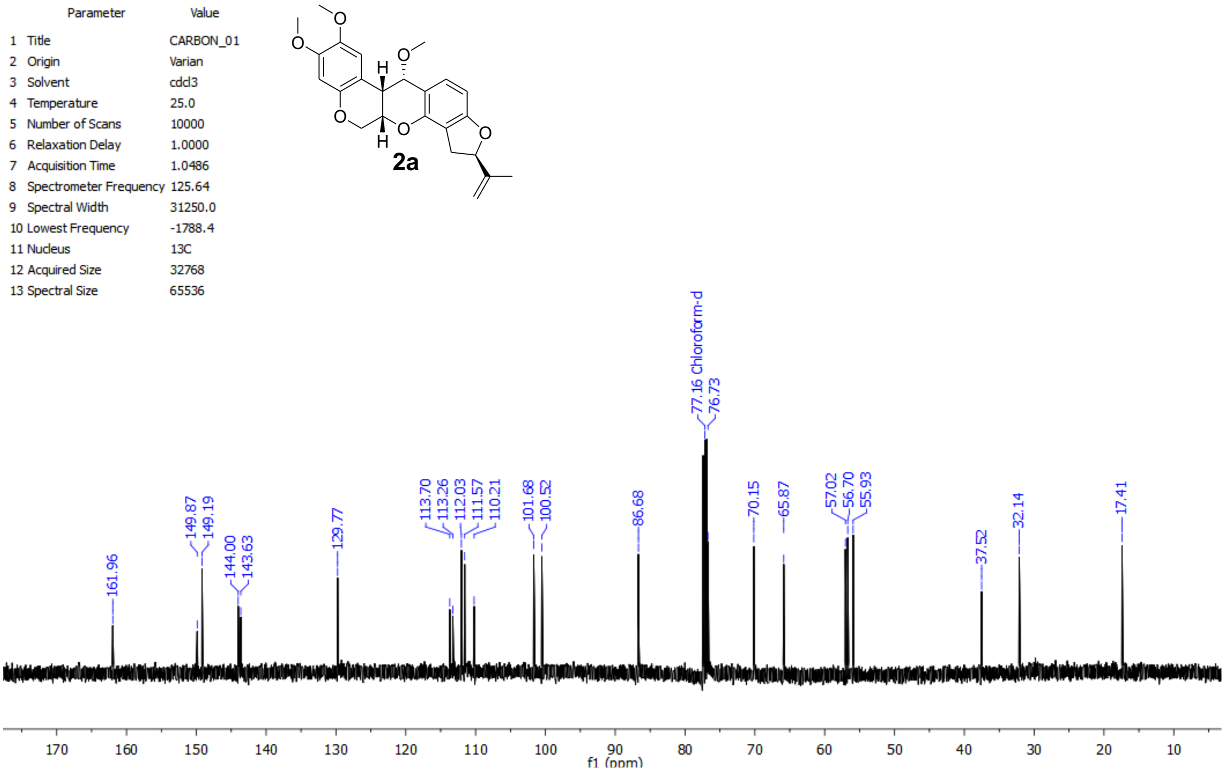

Figure S17. ^{13}C NMR spectra of compound **2a**.

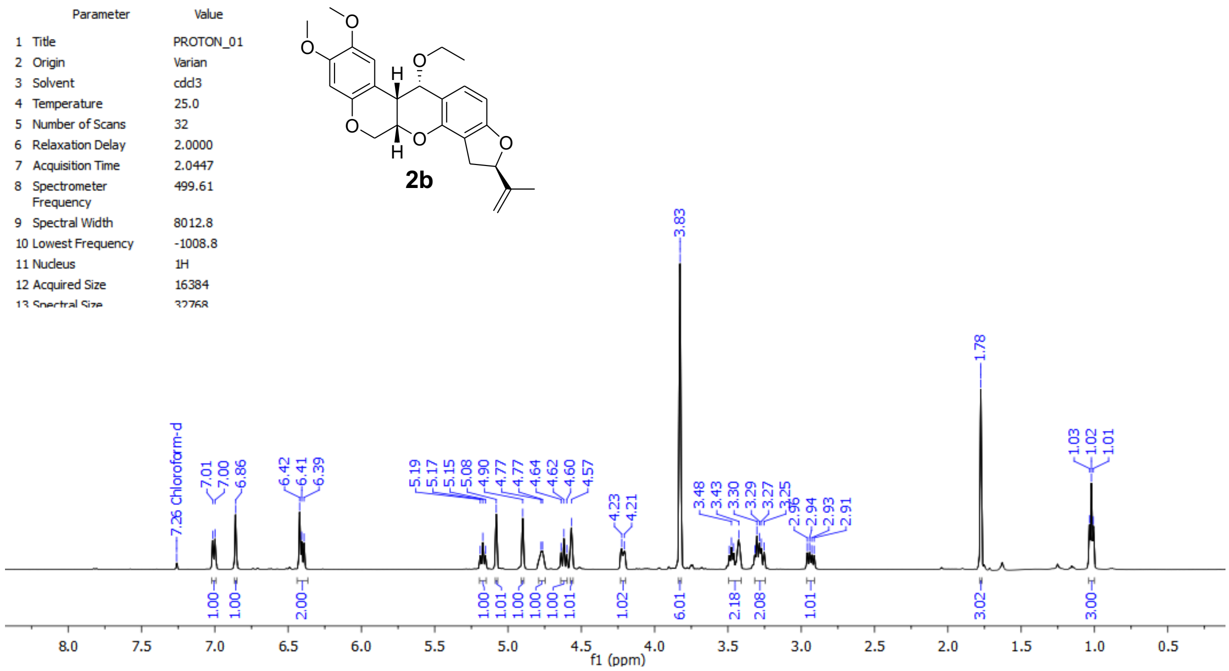

Figure S18. ^{1}H NMR spectra of compound **2b**.

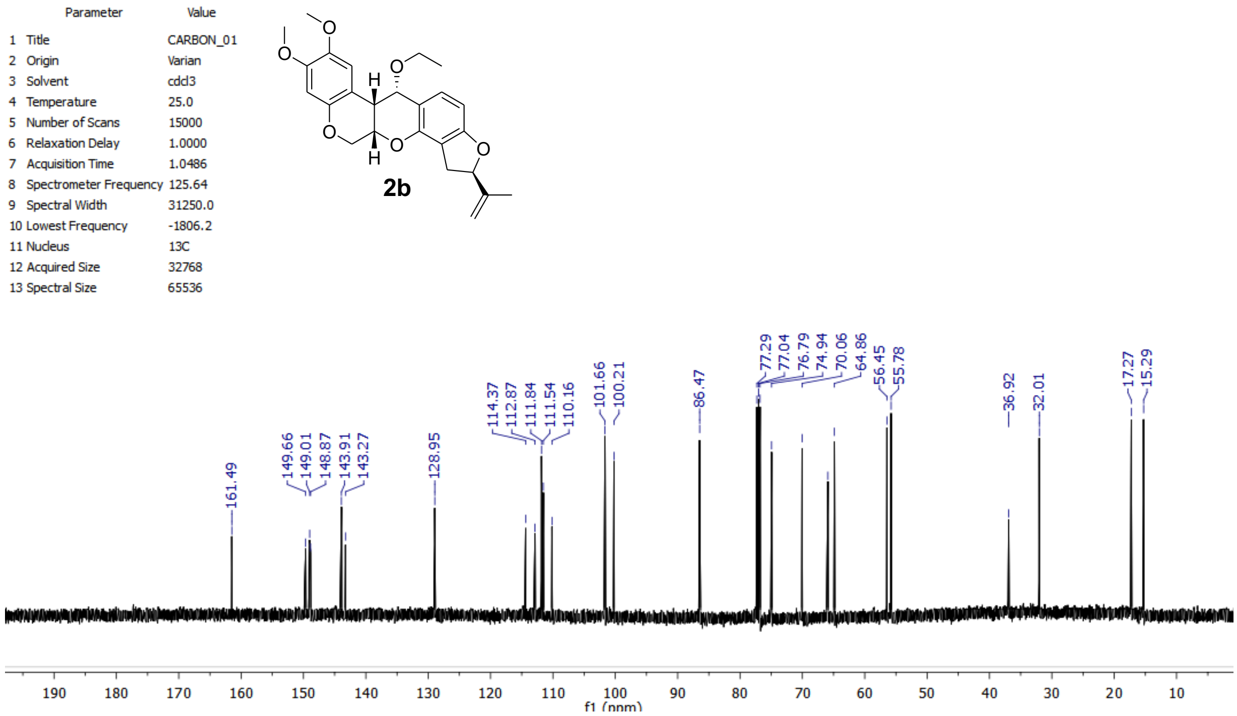

Figure S19. ^{13}C NMR spectra of compound **2b**.

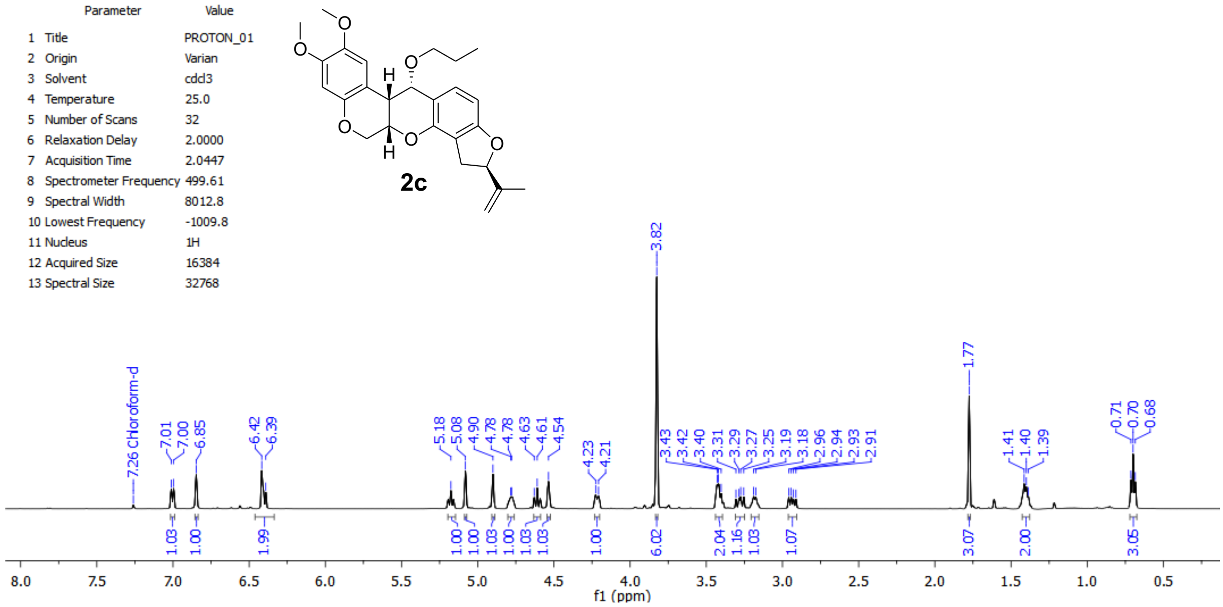

Figure S20. ^{1}H NMR spectra of compound **2c**.

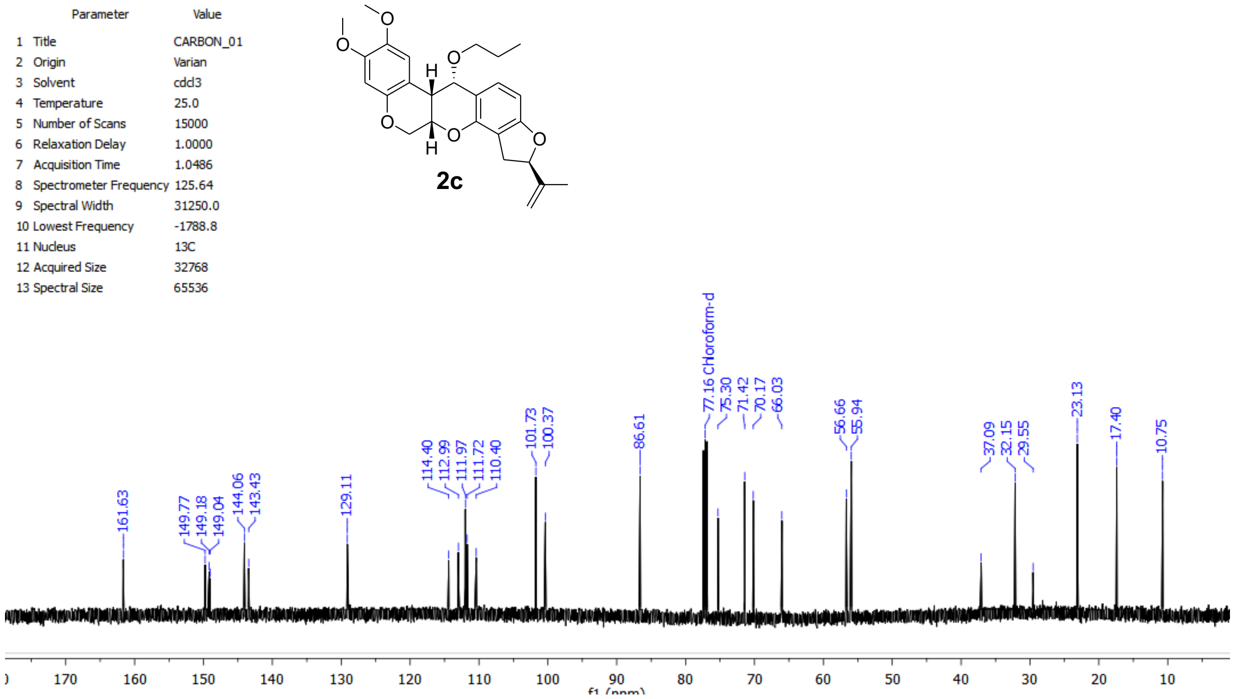

Figure S21. ^{13}C NMR spectra of compound **2c**.

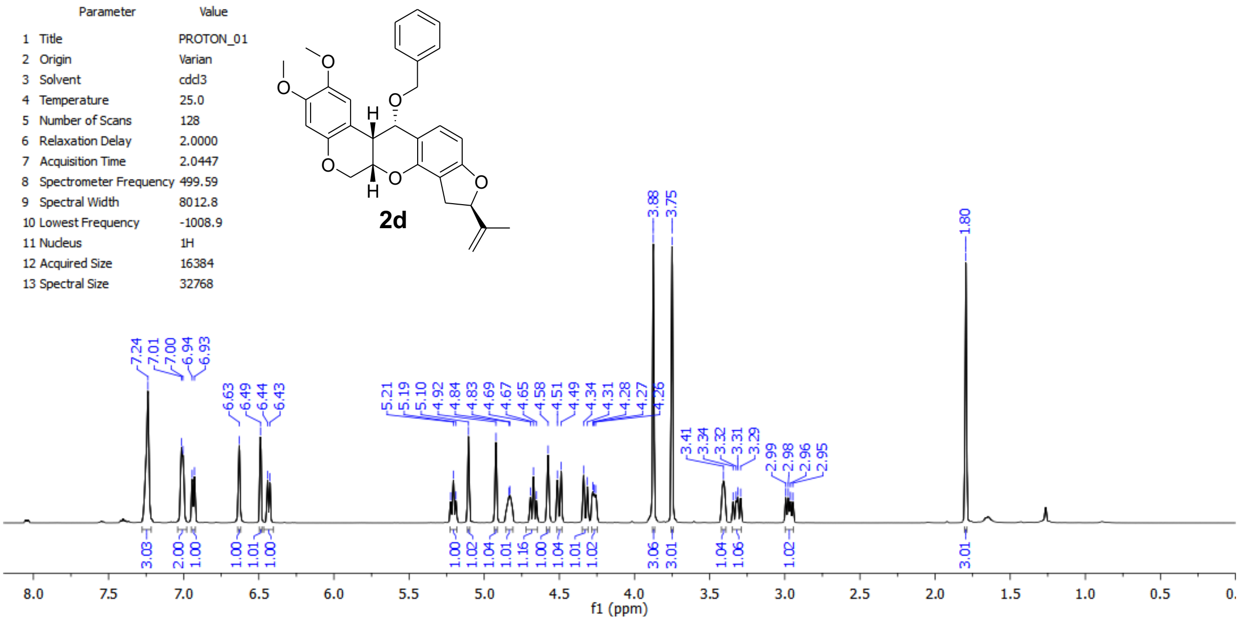

Figure S22. ^{1}H NMR spectra of compound **2d**.

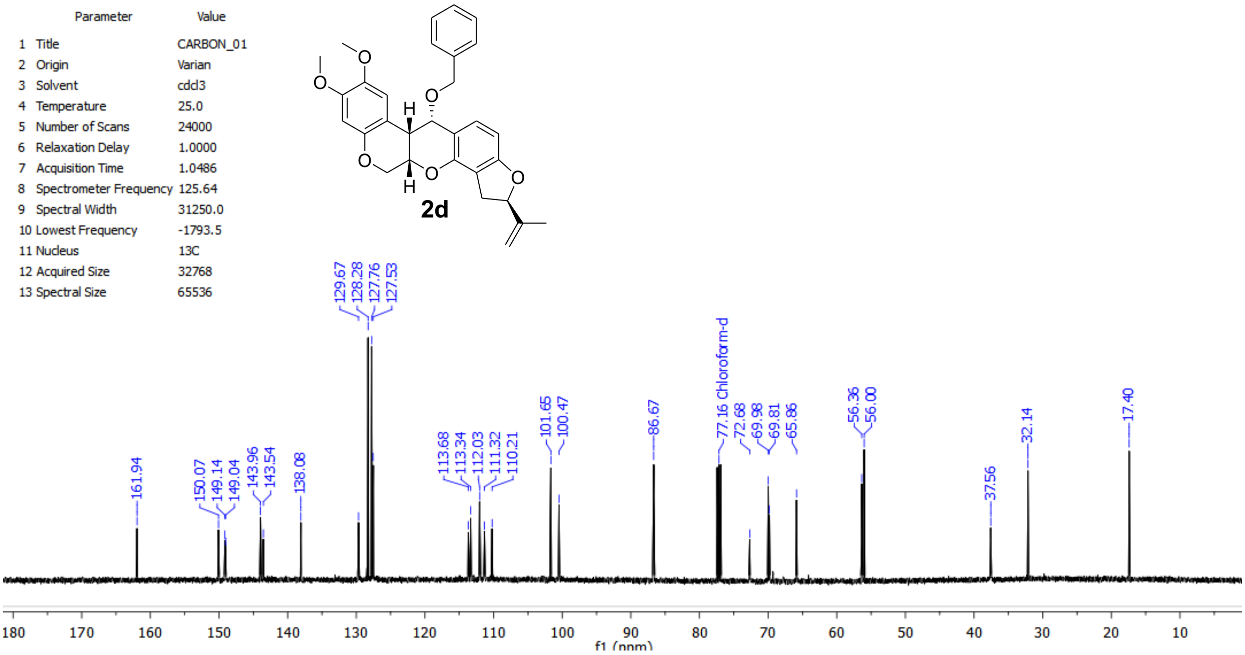

Figure S23. ^{13}C NMR spectra of compound **2d**.

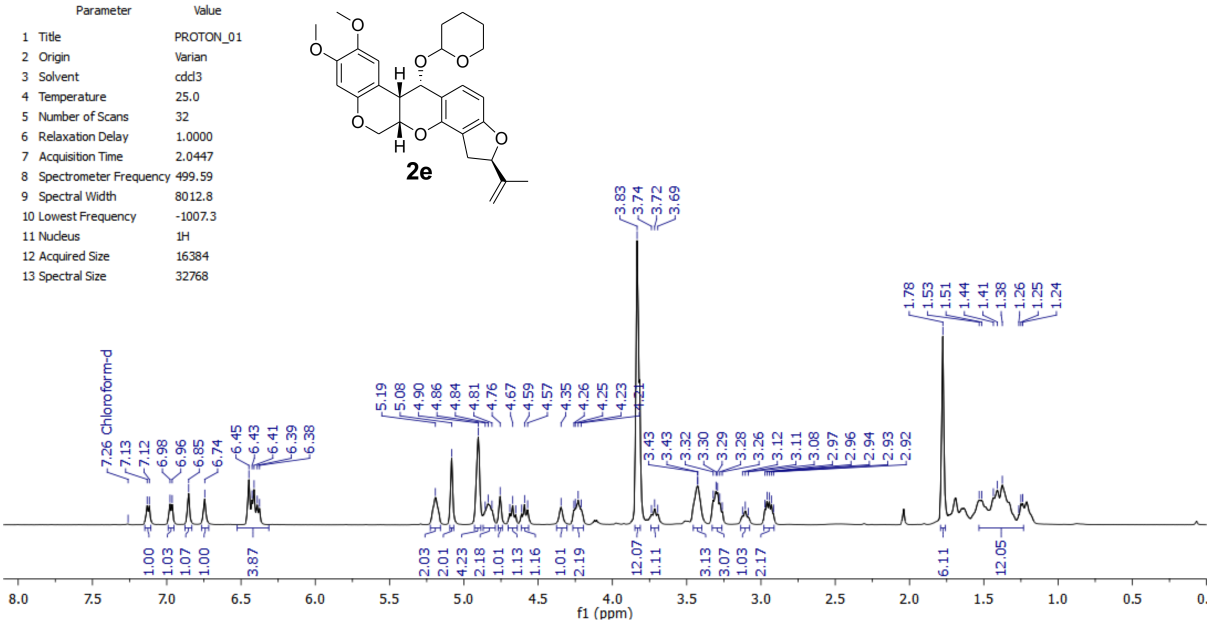

Figure S24. ^{1}H NMR spectra of compound **2e** (mixture of diastereomers).

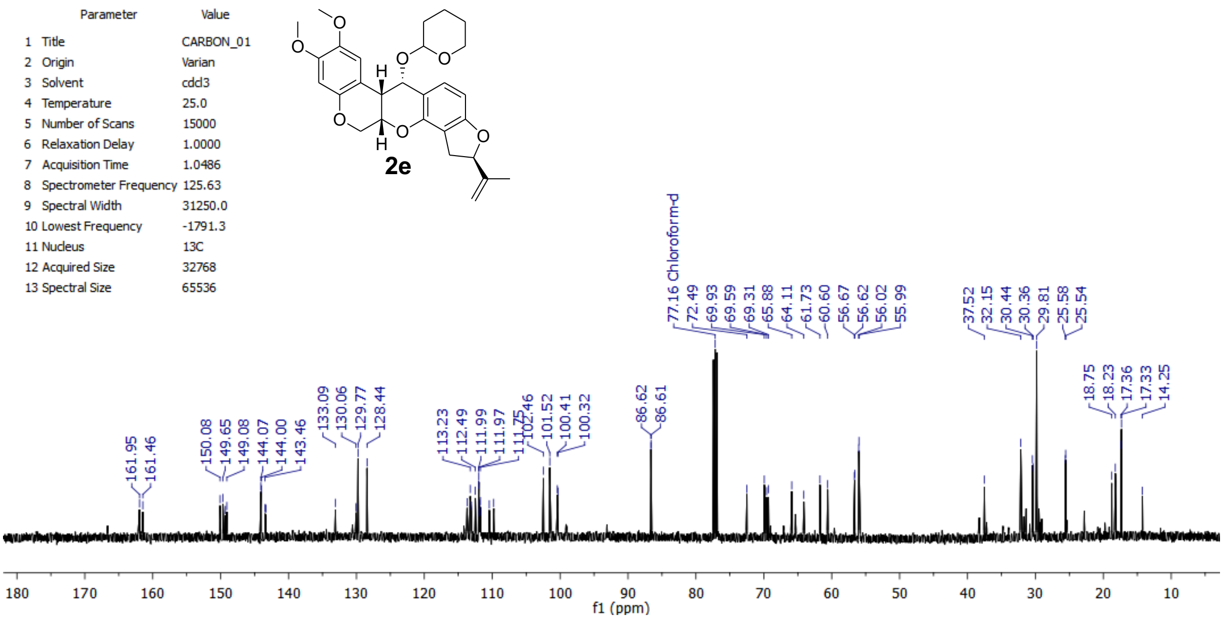

Figure S25. ^{13}C NMR spectra of compound **2e** (mixture of diastereomers).

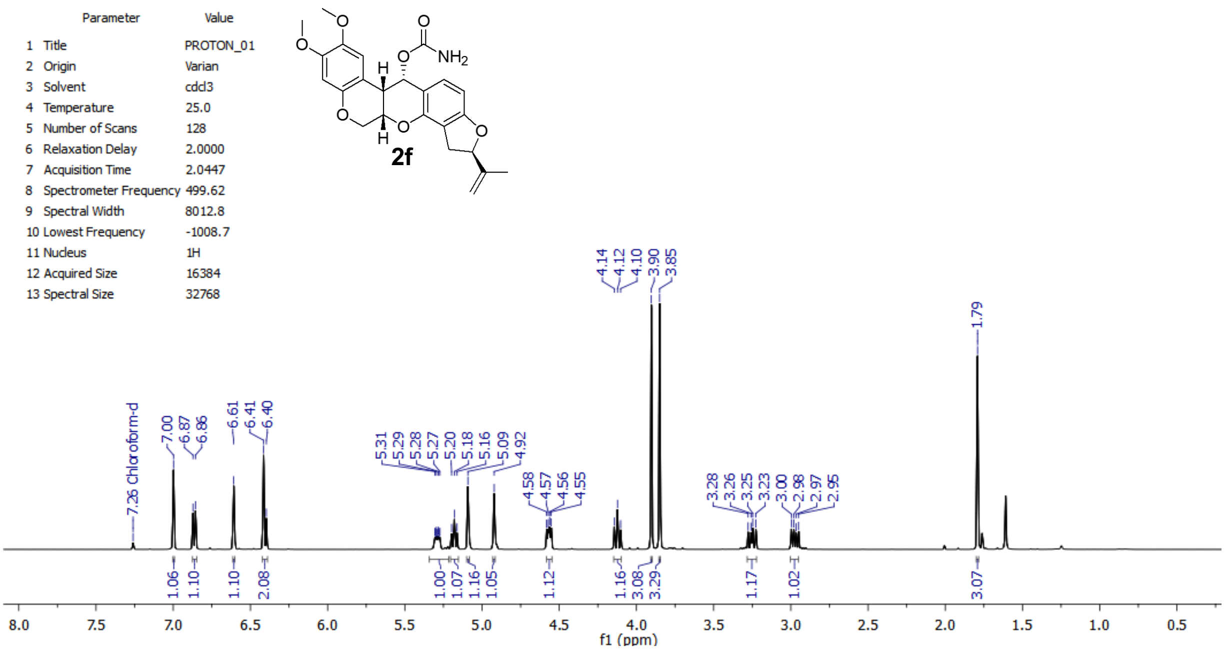

Figure S26. ^{1}H NMR spectra of compound **2f**.

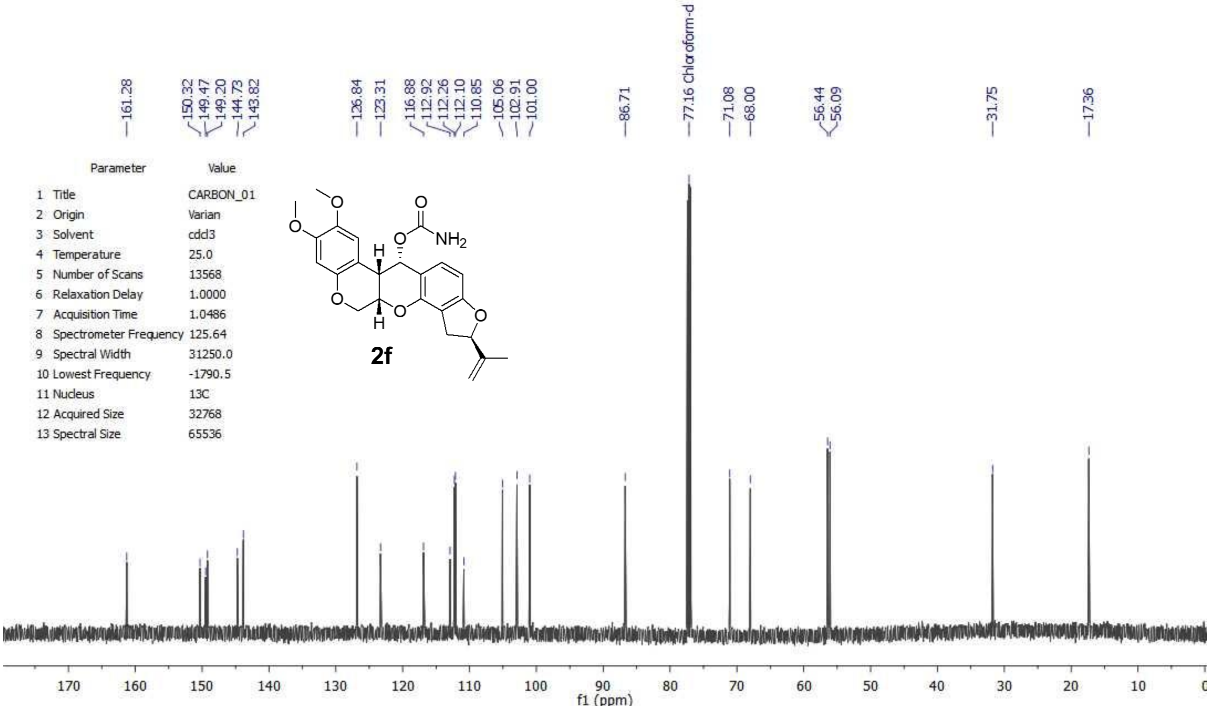

Figure S27. ^{13}C NMR spectra of compound **2f**.

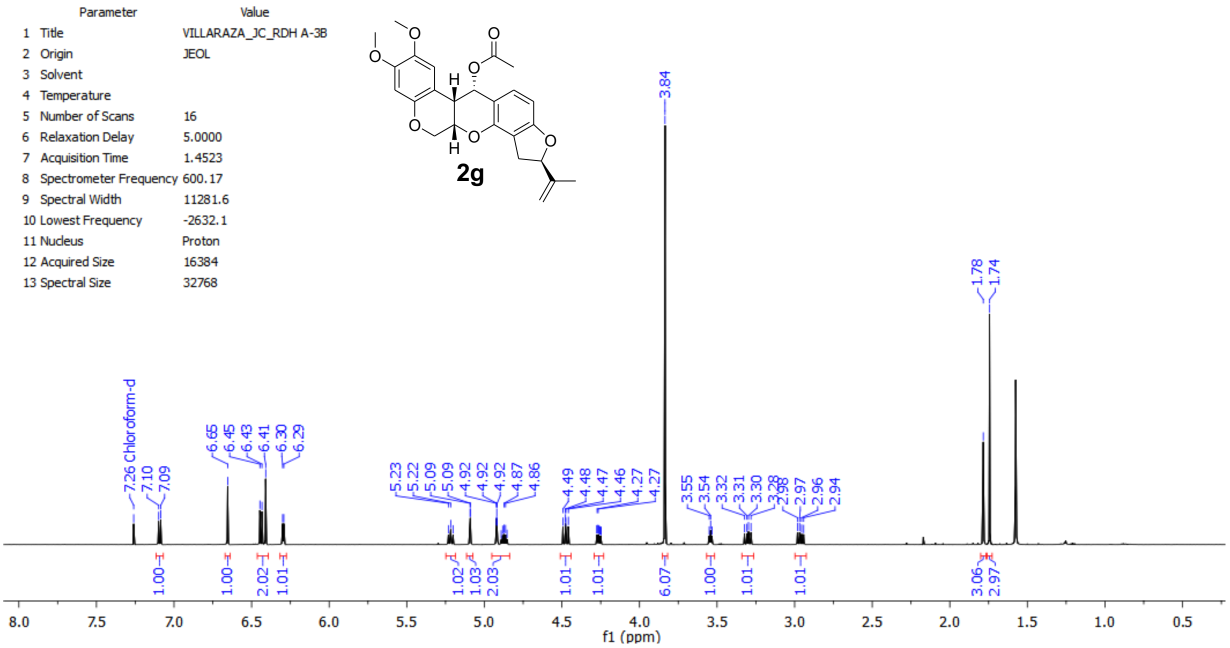

Figure S28. ^{1}H NMR spectra of compound **2g**.

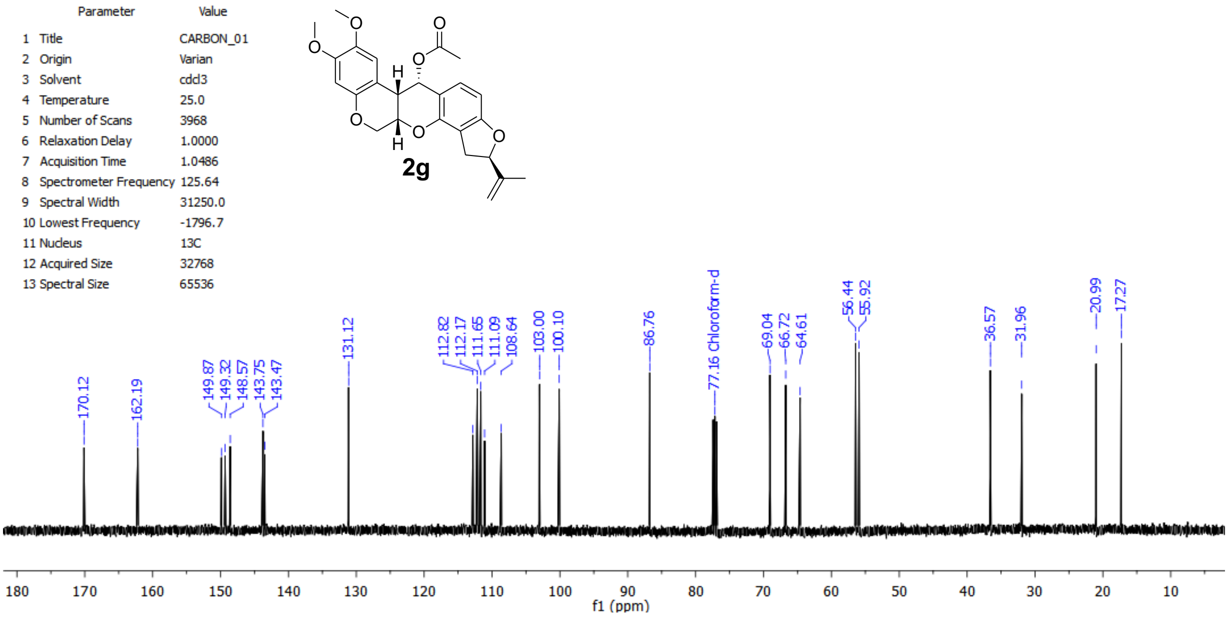

Figure S29. ^{13}C NMR spectra of compound **2g**.

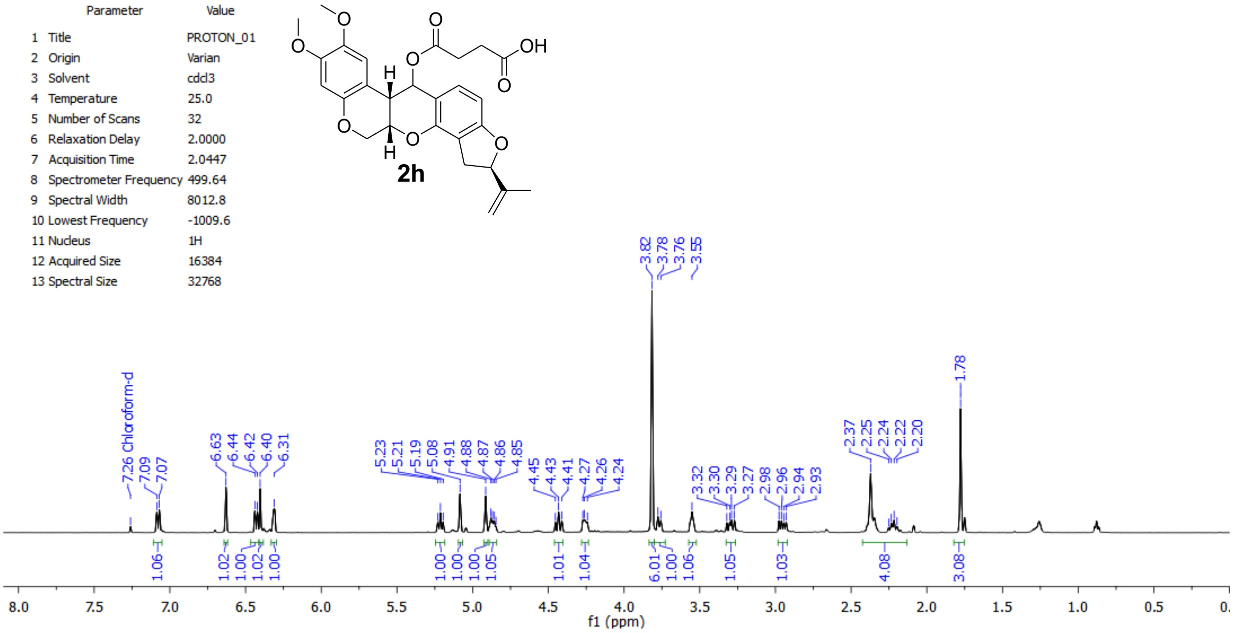

Figure S30. ^{1}H NMR spectra of compound **2h**.

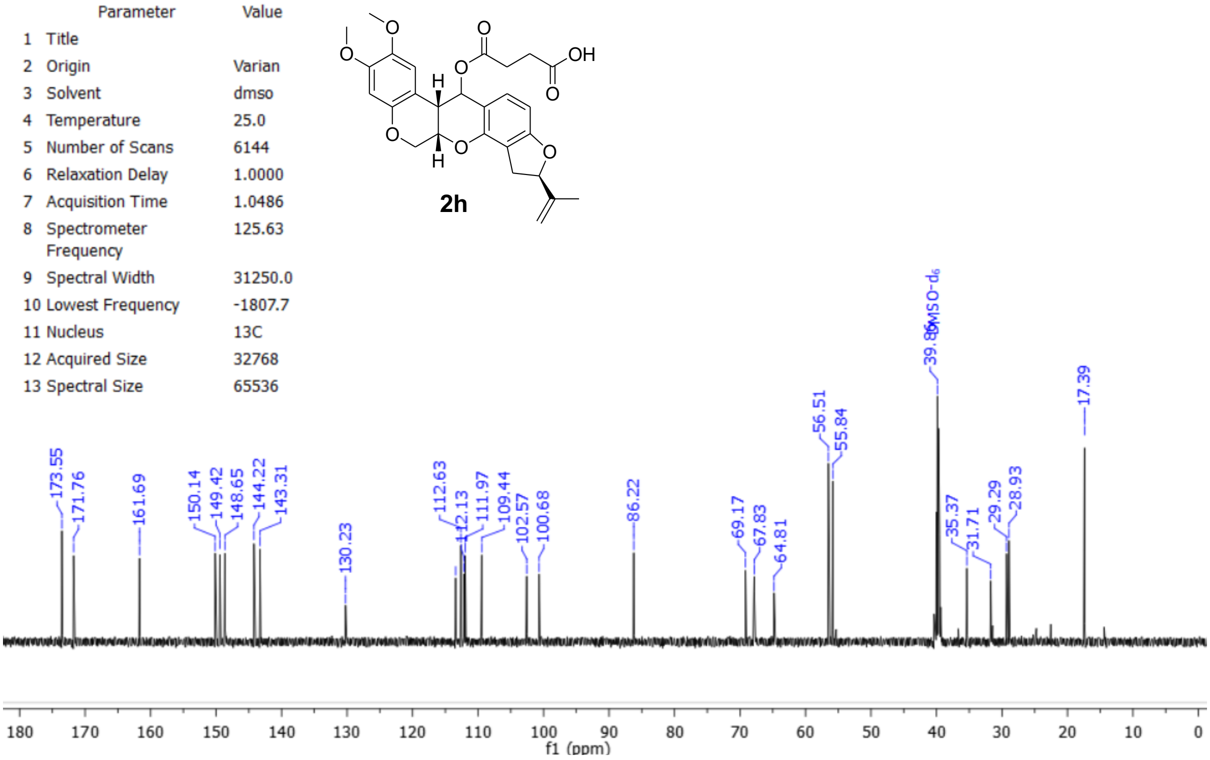

Figure S31. ^{13}C NMR spectra of compound **2h**.

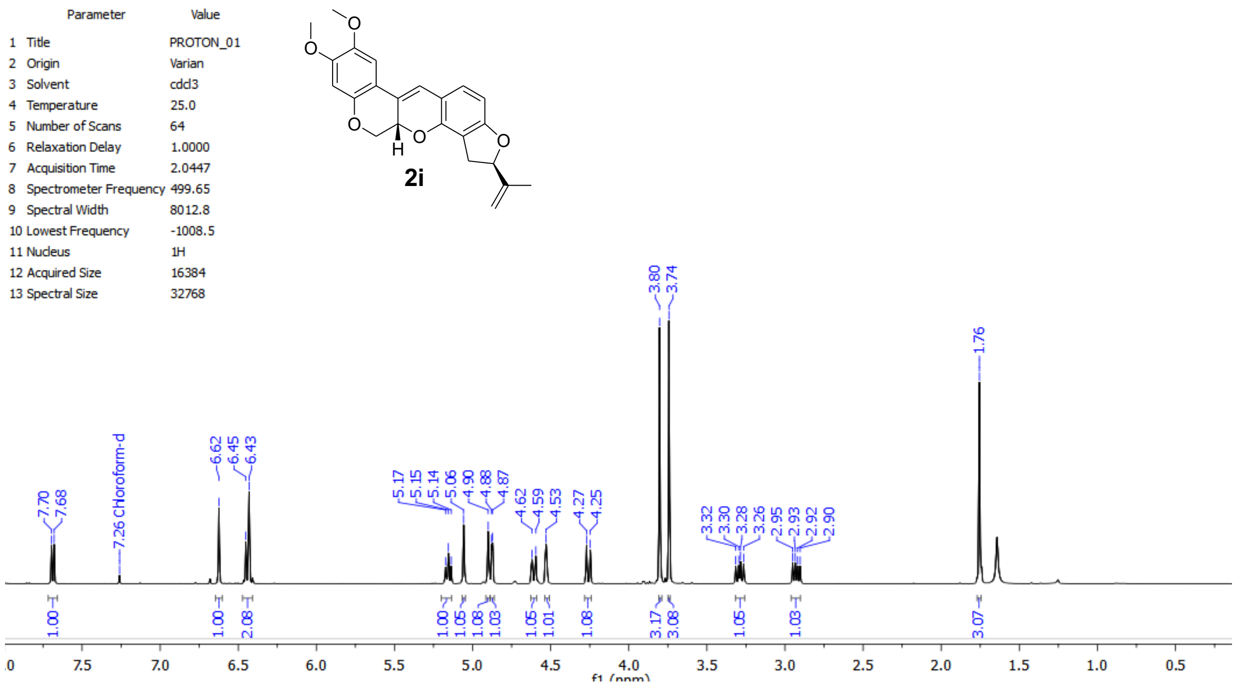

Figure S32. ^{1}H NMR spectra of compound **2i**.

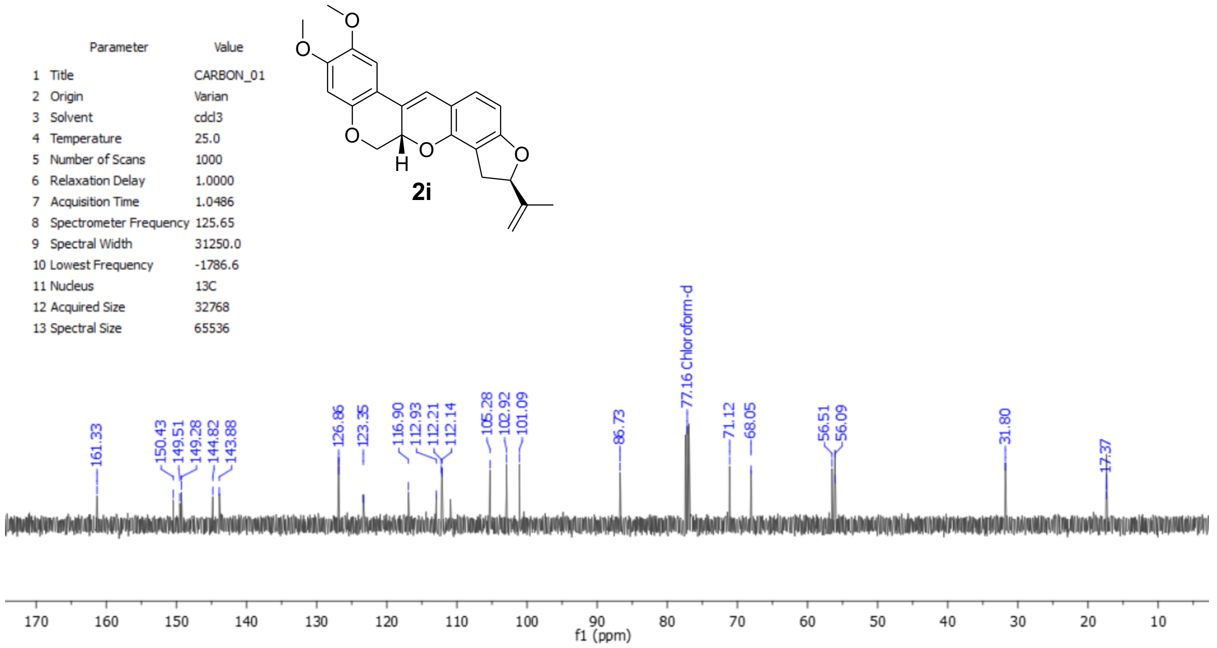

Figure S33. ^{13}C NMR spectra of compound **2i**.

Table S1. IC_{50} values of rotenone derivatives against MCF-7 cancer cells

Compound	MW	MCF-7							
		µg/mL			µM			µM	
		Trial 1	Trial 2	Trial 3	Trial 1	Trial 2	Trial 3	Ave	SD
Doxorubicin	*543.52*	2.42	2.804	4.377	4.452458	5.158964	8.053062	5.89	1.91
Rotenone	*394.41*	<0.78	<0.78	<0.78	1.977637	1.977637	1.977637	1.98	-
**1**	*409.432*	<0.78	<0.78	<0.78	1.905079	1.905079	1.905079	1.91	-
**1a**	*423.168*	n.d.	n.d.	n.d.	-	-	-	-	-
**1b**	*437.184*	n.d.	n.d.	n.d.	-	-	-	-	-
**1c**	*451.2*	>100	>100	>100	>100	>100	>100	>100	-
**1d**	*499.2*	4.32	9.21	10.69	8.653855	18.44954	21.41428	16.17	6.68
**1e**	*451.163*	33.39	32.34	28.27	74.00871	71.68139	62.66027	69.45	5.99
**1f**	*465.179*	18.76	19.05	26.88	40.32858	40.952	57.78423	46.35	9.90
**1g**	*479.194*	26.08	34.71	35.34	54.42468	72.43407	73.74878	66.87	10.80
**2**	*396.433*	<0.78	<0.78	<0.78	1.967545	1.967545	1.967545	1.97	-
**2a**	*410.173*	4.877	5.817	3.299	11.89011	14.18182	8.04295	11.37	3.10
**2b**	*424.189*	2.831	2.651	1.795	6.673918	6.249579	4.231608	5.72	1.30
**2c**	*438.204*	19.19	24.83	28.03	43.79237	56.66308	63.96561	54.81	10.21
**2d**	*486.204*	35.36	37.8	49.78	72.72664	77.74511	102.385	84.29	15.87
**2e**	*480.215*	12.96	11.6	15.54	26.98792	24.15586	32.36052	27.83	4.17
**2f**	*439.163*	>100	>100	>100	>100	>100	>100	>100	-
**2g**	*438.168*	n.d.	n.d.	n.d.	-	-	-	-	-
**2h**	*496.173*	n.d.	n.d.	n.d.	-	-	-	-	-
**2i**	*378.147*	23.48	20.46	34.52	62.0923	54.10599	91.28732	69.16	19.57

n.d. means IC_{50} values were not determined (n.d.) due to low inhibition or compounds did not follow a dose-response curve (i.e., the percent inhibition plateaued at around 40-60% at all concentrations tested)

Table S2. IC_{50} values of rotenone derivatives against A549 cancer cells

Compound	MW	A549							
		µg/mL			µM			µM	
		Trial 1	Trial 2	Trial 3	Trial 1	Trial 2	Trial 3	Ave	SD
Doxorubicin	*543.52*	4.271	3.815	3.908	7.858037	7.019061	7.190168	7.36	0.24
Rotenone	*394.41*	>100	>100	>100	>100	>100	>100	>100	-
**1**	*409.432*	0.11	0.12	0.12	0.268665	0.293089	0.293089	0.28	0.01
**1a**	*423.168*	>100	70.69	98.73	236.3127	167.0494	233.3115	>100	-
**1b**	*437.184*	24.15	23.93	20.65	55.23992	54.7367	47.23414	52.40	4.48
**1c**	*451.2*	>100	>100	>100	>100	>100	>100	>100	-
**1d**	*499.2*	15.48	20.52	n.d.	31.00965	41.10581	-	36.06	7.14
**1e**	*451.163*	53.71	56.86	n.d.	119.0479	126.0298	-	122.54	4.94
**1f**	*465.179*	19.69	16.48	n.d.	42.32781	35.42724	-	38.88	4.88
**1g**	*479.194*	45.59	50.18	20.67	95.13884	104.7174	43.13489	81.00	33.14
**2**	*396.433*	0.17	0.2	0.17	0.428824	0.504499	0.428824	0.45	0.04
**2a**	*410.173*	>100	>100	>100	>100	>100	>100	>100	-
**2b**	*424.189*	29.17	25.94	29.35	68.76658	61.15204	69.19092	66.37	4.52
**2c**	*438.204*	>100	>100	>100	>100	>100	>100	>100	0.00
**2d**	*486.204*	64.22	28.73	46.84	132.0844	59.0904	96.33812	95.84	36.50
**2e**	*480.215*	20.73	12.07	11.69	43.16818	25.13459	24.34327	30.88	10.65
**2f**	*439.163*	>100	>100	>100	>100	>100	>100	>100	-
**2g**	*438.168*	>100	>100	>100	>100	>100	>100	>100	-
**2h**	*496.173*	>100	>100	>100	>100	>100	>100	>100	-
**2i**	*378.147*	0.05	0.04	0.04	0.132224	0.105779	0.105779	0.11	0.02

n.d. means IC_{50} values were not determined (n.d.) due to low inhibition or compounds did not follow a dose-response curve (i.e., the percent inhibition plateaued at around 40-60% at all concentrations tested)

Table S3. IC_{50} values of rotenone derivatives against HCT116 cancer cells

Compound	MW	HCT116							
		µg/mL			µM			µM	
		Trial 1	Trial 2	Trial 3	Trial 1	Trial 2	Trial 3	Ave	SD
Doxorubicin	*543.52*	0.13	0.1142	0.09641	0.239182	0.210112	0.177381	0.21	0.03
Rotenone	*394.41*	0.78	0.71	1.06	1.977637	1.800157	2.687559	2.16	0.47
**1**	*409.432*	0.15	0.21	0.52	0.366361	0.512906	1.270053	0.72	0.49
**1a**	*423.168*	>100	>100	>100	>100	>100	>100	>100	-
**1b**	*437.184*	10.7	6.498	5.984	24.47483	14.86331	13.68761	17.68	5.92
**1c**	*451.2*	>100	>100	>100	>100	>100	>100	>100	-
**1d**	*499.2*	25.99	n.d.	n.d.	52.06335	-	-	-	-
**1f**	*451.163*	25.2	15.23	24.87	55.85563	33.75719	55.12419	48.25	12.55
**1f**	*465.179*	23.88	n.d.	30.28	51.3351	-	65.09325	25.67	36.30
**1g**	*479.194*	n.d.	n.d.	n.d.	-	-	-	-	-
**2**	*396.433*	1.24	1.49	2.01	3.127892	3.758516	5.070212	3.99	0.99
**2a**	*410.173*	46.28	*>100*	62.26	112.8305	243.7996	151.7896	132.31	27.55
**2b**	*424.189*	16.75	21.16	17.06	39.48715	49.88347	40.21796	43.20	5.80
**2c**	*438.204*	53.36	48.94	38.78	121.7697	111.6831	88.49755	107.32	17.06
**2d**	*486.204*	59.93	55.37	30.57	123.261	113.8822	62.87482	100.01	32.50
**2e**	*480.215*	>100	>100	>100	>100	>100	>100	>100	-
**2f**	*439.163*	3.894	3.89	3.886	8.866865	8.857757	8.848649	8.86	0.01
**2g**	*438.168*	>100	>100	>100	>100	>100	>100	>100	-
**2h**	*496.173*	>100	>100	>100	>100	>100	>100	>100	-
**2i**	*378.147*	20.26	19.12	9.69	53.57709	50.56239	25.62498	43.25	15.34

n.d. means IC_{50} values were not determined (n.d.) due to low inhibition or compounds did not follow a dose-response curve (i.e., the percent inhibition plateaued at around 40-60% at all concentrations tested)

Table S4. Pharmacokinetic Parameters of Rotenone and Its Derivatives

Molecule	GI absorption	BBB permeant	Pgp substrate
**Rotenone**	High	Yes	No
**1**	High	No	No
**1a**	High	Yes	No
**1b**	High	Yes	No
**1c**	High	Yes	No
**1d**	High	No	Yes
**1e**	High	No	No
**1f**	High	No	No
**1g**	High	No	Yes
**2**	High	Yes	No
**2a**	High	Yes	No
**2b**	High	Yes	No
**2c**	High	Yes	No
**2d**	High	Yes	No
**2e**	High	Yes	No
**2f**	High	No	No
**2g**	High	Yes	No
**2h**	High	No	No
**2i**	High	Yes	No

Table S5. Drug likeness Rules Score and Medicinal Chemistry Prediction of Rotenone and Its Derivatives

Molecule	Lipinski	Ghose	Veber	Egan	Muegge	Bioavailability Score	PAINS
**Rotenone**	Yes; 0 violation	Yes	Yes	Yes	Yes	0.55	0 alert
**1**	Yes; 0 violation	Yes	Yes	Yes	Yes	0.55	0 alert
**1a**	Yes; 0 violation	Yes	Yes	Yes	Yes	0.55	0 alert
**1b**	Yes; 0 violation	Yes	Yes	Yes	No; 1 violation: XLOGP3>5	0.55	0 alert
**1c**	Yes; 0 violation	Yes	Yes	Yes	No; 1 violation: XLOGP3>5	0.55	0 alert
**1d**	Yes; 0 violation	No; 2 violations: MW>480, MR>130	Yes	Yes	No; 1 violation: XLOGP3>5	0.55	0 alert
**1e**	Yes; 0 violation	Yes	Yes	Yes	Yes	0.55	0 alert
**1f**	Yes; 0 violation	Yes	Yes	Yes	Yes	0.55	0 alert
**1g**	Yes; 0 violation	Yes	Yes	Yes	No; 1 violation: XLOGP3>5	0.55	0 alert
**2**	Yes; 0 violation	Yes	Yes	Yes	Yes	0.55	0 alert
**2a**	Yes; 0 violation	Yes	Yes	Yes	Yes	0.55	0 alert
**2b**	Yes; 0 violation	Yes	Yes	Yes	Yes	0.55	0 alert
**2c**	Yes; 0 violation	Yes	Yes	Yes	No; 1 violation: XLOGP3>5	0.55	0 alert
**2d**	Yes; 0 violation	No; 2 violations: MW>480, MR>130	Yes	Yes	No; 1 violation: XLOGP3>5	0.55	0 alert
**2e**	Yes; 0 violation	No; 2 violations: MW>480, MR>130	Yes	Yes	Yes	0.55	0 alert
**2f**	Yes; 0 violation	Yes	Yes	Yes	Yes	0.55	0 alert
**2g**	Yes; 0 violation	Yes	Yes	Yes	Yes	0.55	0 alert
**2h**	Yes; 0 violation	No; 1 violation: MW>480	Yes	Yes	Yes	0.56	0 alert
**2i**	Yes; 0 violation	Yes	Yes	Yes	Yes	0.55	0 alert

Present affiliation of Jannelle R. Casanova is Institut für Biochemie, Universität Greifswald, Greifswald, Germany 17489
